# *SCGN* deficiency is a risk factor for autism spectrum disorder

**DOI:** 10.1038/s41392-022-01225-2

**Published:** 2023-01-02

**Authors:** Zhe Liu, Shuai Tan, Lianyu Zhou, Li Chen, Mingfeng Liu, Wang Wang, Yingying Tang, Qin Yang, Sensen Chi, Peiyan Jiang, Yue Zhang, Yonghua Cui, Junhong Qin, Xiao Hu, Shenglong Li, Qi Liu, Lu Chen, Song Li, Ezra Burstein, Wei Li, Xiaohu Zhang, Xianming Mo, Da Jia

**Affiliations:** 1 Key Laboratory of Birth Defects and Related Diseases of Women and Children, Department of Paediatrics, West China Second University Hospital, State Key Laboratory of Biotherapy and Collaborative Innovation Center of Biotherapy, Sichuan University, 610041 Chengdu, China; 2 Department of Immunology, College of Basic Medicine, Chongqing Medical University, 400010 Chongqing, China; 3 Department of Neurosurgery, Xinqiao Hospital, Army Medical University, Chongqing, China; 4 Beijing Key Laboratory for Genetics of Birth Defects, Beijing Pediatric Research Institute, 100045 Beijing, China; 5 MOE Key Laboratory of Major Diseases in Chidren, Beijing Children’s Hospital, Capital Medical University, 100045 Beijing, China; 6 Department of Psychology, Beijing Children’s Hospitl, Capital Medical University, 100045 Beijing, China; 7 Department of Internal Medicine, University of Texas Southwestern Medical Center, Dallas, TX 75390 USA; 8 Sichuan University-The Chinese University of Hong Kong Joint Laboratory for Reproductive Medicine, West China Second University Hospital, Sichuan University, Chengdu, China; 9 Department of Pediatric Surgery and Laboratory of Stem Cell Biology, State Key Laboratory of Biotherapy, West China Hospital, Sichuan University, 610041 Chengdu, China

**Keywords:** Neurodevelopmental disorders, Diseases of the nervous system

## Abstract

Autism spectrum disorder (ASD) affects 1–2% of all children and poses a great social and economic challenge for the globe. As a highly heterogeneous neurodevelopmental disorder, the development of its treatment is extremely challenging. Multiple pathways have been linked to the pathogenesis of ASD, including signaling involved in synaptic function, oxytocinergic activities, immune homeostasis, chromatin modifications, and mitochondrial functions. Here, we identify secretagogin (*SCGN*), a regulator of synaptic transmission, as a new risk gene for ASD. Two heterozygous loss-of-function mutations in *SCGN* are presented in ASD probands. Deletion of *Scgn* in zebrafish or mice leads to autism-like behaviors and impairs brain development. Mechanistically, *Scgn* deficiency disrupts the oxytocin signaling and abnormally activates inflammation in both animal models. Both ASD probands carrying *Scgn* mutations also show reduced oxytocin levels. Importantly, we demonstrate that the administration of oxytocin and anti-inflammatory drugs can attenuate ASD-associated defects caused by SCGN deficiency. Altogether, we identify a convergence between a potential autism genetic risk factor *SCGN*, and the pathological deregulation in oxytocinergic signaling and immune responses, providing potential treatment for ASD patients suffering from SCGN deficiency. Our study also indicates that it is critical to identify and stratify ASD patient populations based on their disease mechanisms, which could greatly enhance therapeutic success.

## Introduction

Autism spectrum disorder (ASD) is a heterogeneous group of neurodevelopmental diseases characterized by deficits in communication and social interaction.^1,2^ Most cases are diagnosed before the age of two; however, certain ASD-associated symptoms can appear later in life.^3^ Autistic individuals display certain core behavioral features such as impairments in communication and social interaction, and repetitive or restricted sensory–motor behaviors.^4^ In addition to these core symptoms, autistic individuals can develop delayed development, intellectual disability, anxiety, depression, and gastrointestinal and immune conditions.^5^ It is estimated that ASD affects 1–2% of all children, causing significant social and economic burdens for society.^6^ The heterogeneous clinical features of ASD have greatly challenged the development of effective treatments. Two antipsychotic drugs, risperidone and aripiprazole, are the only FDA-approved drugs for ASD patients;^7^ however, both have limited effects and the potential for significant side effects. Further understanding of the mechanisms of pathogenesis will be indispensable for the development of novel therapeutic strategies for ASD.

The variable phenotypical presentations of ASD are mirrored by its complex causes. Genetic, prenatal, perinatal, and environmental factors may collectively contribute to ASD development. Nonetheless, 74~93% of ASD risk is estimated to be heritable.^8–10^ So far, over 1000 ASD candidate genes have been included in the SFARI (Simons Foundation Autism Research Initiative) database (https://gene.sfari.org/autdb/GS_Home.do). However, the pathological mechanisms of these genes remain largely uncovered. In recent years, studies including genome-wide sequencing technologies have revealed a large set of ASD-risk genes,^11–14^ which are often associated with a number of biological functions/pathways, including synaptic structure and functions (e.g., SNAP-25, STXBP2, SHANK3, and NLGN3), chromatin modification (e.g., CHD7 and MECP2), Wnt signaling (e.g., CHD8, PAX5, and ATRX), oxytocinergic signaling, immune function, and mitochondrial function, among other processes.^2,15–17^ One emerging concept in the field is that these pathways could converge on the regulation of a few key functional activities. For instance, the loss of synaptic adhesion molecule Nlgn3 impairs oxytocin signaling and disrupts normal translation. Conversely, pharmacologic treatment of Nlgn3-knockout mice resets the translation homeostasis and restores oxytocin responses.^18^ Therefore, dissecting the functions of ASD-risk genes may be essential to understand the heterogeneity of autism, and knowledge of their common and unique underlying mechanisms may help to develop therapeutic strategies with improved efficacy.

Secretagogin (*SCGN*) is a gene conserved from fruit flies to humans and is expressed in multiple endocrine organs, including the pancreas, the gastrointestinal tract, the thyroid, the adrenal medulla, the adrenal gland, and the brain.^19,20^ It is highly enriched in pancreatic β-cells, where it controls insulin secretion^21^ and is similarly expressed by a subset of enteroendocrine cells in the gut,^19^ where it also facilitates hormone release. In the human brain, *SCGN* is highly expressed in the hippocampus and cerebellum, where it mediates the secretion of multiple proteins, including matrix metalloproteinase-2 (MMP2) and corticotropin-releasing hormone (CRH).^22,23^ *SCGN* encodes a calcium-sensing protein that interacts with the SNARE core component SNAP-25 in a Ca^2+^-dependent manner to regulate membrane fusion events.^24^ In our recent study, we reported the crystal structures of SCGN in complex with SNAP-25, and showed that *scgn* was critical for neuronal development in zebrafish.^25^ SCGN deficiency has been linked to the pathogenesis of various disorders, including diabetes, schizophrenia and neurodegeneration, and more recently, autosomal recessive early-onset ulcerative colitis.^26–30^ However, the molecular functions of SCGN in the nervous system and its connection with neurological disorders are still unclear.

In this study, two heterozygous mutations in *SCGN* are identified in ASD probands. We show that these disease-causing mutations result in the loss of SCGN functions. Using both zebrafish and mouse model systems, we show that SCGN deficiency leads to autistic behaviors via disrupting activating pro-inflammatory responses and the oxytocinergic signaling in the brain. Treatment of SCGN-deficient animals with anti-inflammatory drugs and oxytocin analogs can correct autistic behaviors. Our study not only identifies a novel ASD-risk gene but also presents mechanism-based interventions with potential applicability in the treatment of ASD.

## Results

### Identification of novel *SCGN* mutations in two ASD probands

**Fig. 1:**
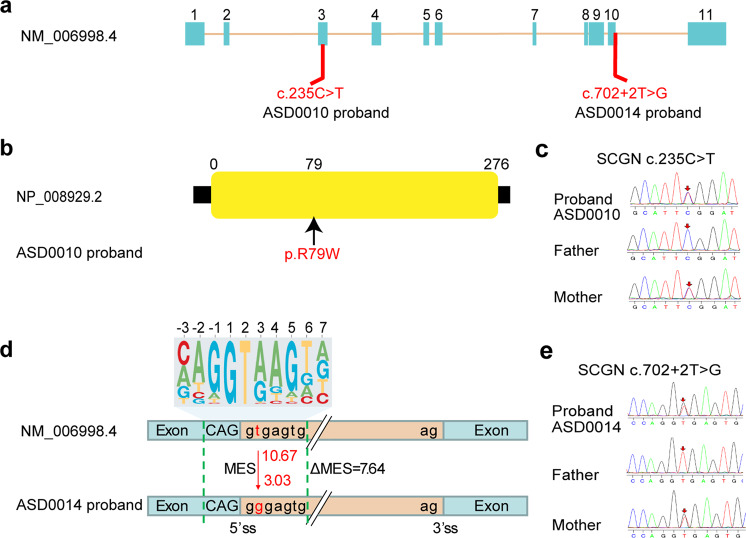
Mutations in *SCGN* are linked to ASD. **a** Schematic diagram of mutation sites in two ASD probands. **b** ASD0010 carries one heterozygous, missense mutation (R79W) in the *SCGN* gene. **c** Sanger sequencing traces of unaffected parents and affected ASD0010 proband. **d** ASD0014 carries a heterozygous mutation in *SCGN* immediately after exon 10, which likely affect gene splicing. 10.67 and 3.03 are predicted 5’ splice site score for WT and the mutation. The top panel is the 5’ splicing site motif. MES Maximum Entropy Score. **e** Sanger sequencing traces of unaffected parents and affected ASD0014 proband

To identify potential ASD-risk genes in a Chinese cohort, we performed whole-exome sequencing of our ASD probands and their parents. All individuals with ASD were diagnosed based on the Childhood Autism Rating Scale (CARS), the Autism Behavior Checklist (ABC), the Clancy Autism Behavior Scale (CABS), and the Developmental Quotient (DQ) tests. Among 168 cases that we have sequenced, two individuals were identified with mutations in *SCGN*. One male child, designated as ASD0010, carried one heterozygous mutation (NM006998.4:c.235 C > T:p.R79W) inherited from his mother, which resulted in a missense mutation in *SCGN* (Fig. 1a–c). The other patient, designated as ASD0014, was also a male child carrying a different heterozygous mutation in *SCGN* (NM006998.4:c.702 + 2 T > G) inherited from his mother, as validated by Sanger sequencing (Fig. 1a, d, e). Bioinformatic analysis predicted that this mutation significantly altered the 5’ splicing activity of *SCGN* at a site immediately after exon 10 (splicing score from 10.67 to 3.03 by MaxEntScan,^31^) which retained the following intron and might subsequently lead to protein degradation or loss of function. Indeed, a minigene splicing assay demonstrated that the mutation identified from ASD0014 significantly altered the normal splicing of *SCGN* (Supplementary Fig. 1). We confirmed that both patients exhibited typical core ASD features, including defects in communication and social interaction, measured by CABS, ABC, and CARS scores (Supplementary Table 1). Additionally, both ASD0010 and ASD0014 showed profound developmental delay, as measured by the Gesell development scale (Supplementary Table 1). However, no obvious symptoms of ASD were detected in the probands’ mothers, although they carry the same SCGN mutations. Thus, mutations in SCGN may represent a risk factor for ASD that predominately impacts males (see Discussion).

### *Scgn* deficiency leads to autism-like behaviors in zebrafish

To determine the role of SCGN in social behavior and neuronal development, we generated zebrafish and mouse models with *Scgn*-deletion using CRISPR/Cas9 gene editing technology. Remarkably, *scgn* knockout in both animal models exhibited shared phenotypes. We focus on our zebrafish data and discuss some common findings of mouse and zebrafish models here.

**Fig. 2:**
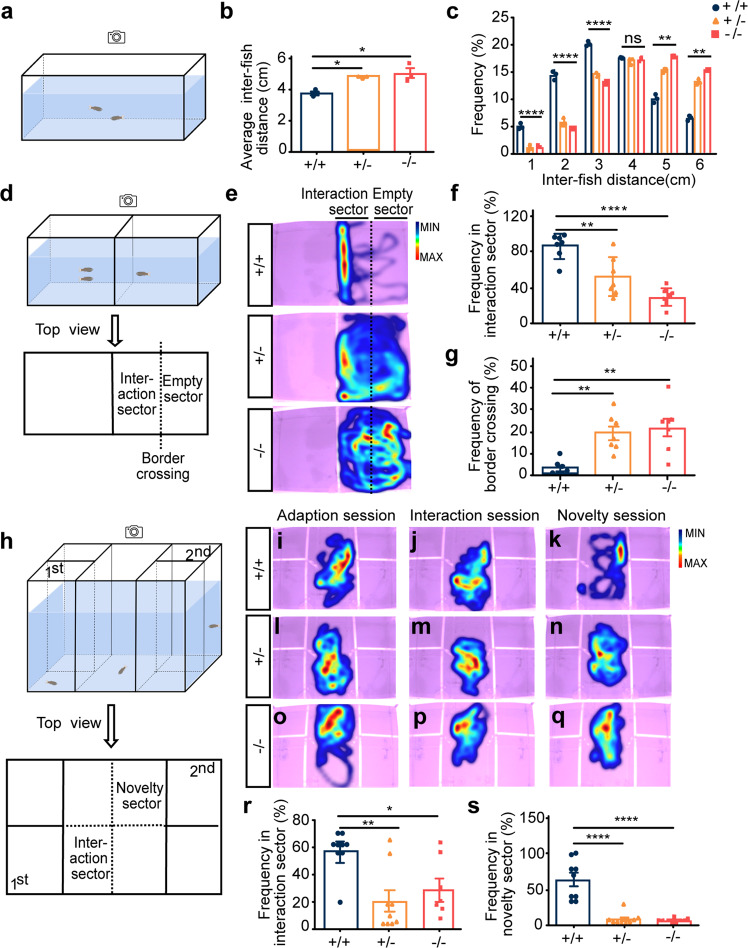
*Scgn*^+/−^ and *scgn*^−/−^ zebrafish show autistic-like behavior. **a** Schematic diagram of shoaling test. Inter-fish distance is recorded; **b** S*cgn*^+/−^ and *scgn*^−/−^ adult zebrafish (4 month old) displayed a significantly increased-fish distance in shoaling test, relative to their wild-type siblings. +/+:*scgn*^+/+^; +/−:*scgn*^+/−^; −/−:*scgn*^−/−^. *p < 0.05. *P-*values were calculated using one-way ANOVA, Tukey’s multiple comparisons test. **c** Frequency at different inter-fish distance in shoaling test. +/+:*scgn*^+/+^; +/−:*scgn*^+/−^; −/−:*scgn*^−/−^. ****p < 0.0001, ***p < 0.001, **p < 0.01, *p < 0.05. ns not significant. *P*-values were calculated using one-way ANOVA, Tukey’s multiple comparisons test. **d** Schematic diagram of two-sector social interaction test. Two stimulus zebrafish were placed in the left chamber. Border crossing is defined as moving from the interaction sector to the empty sector, or vice versa. **e** Heatmaps show that *scgn*^+/+^ zebrafish displayed remarkable higher frequency close to the stimulus zebrafish than *scgn*^+/−^ and *scgn*^−/−^ zebrafish. Warmer colors (red) indicate a greater amount of time spent exploring by the zebrafish. **f** Frequency of test fish in the interaction sector. ****p < 0.0001, **p < 0.01. *P*-values were calculated using one-way ANOVA, Tukey’s multiple comparisons test. **g** Frequency of border crossing between the interaction and the empty sectors. **p < 0.01. *P*-values were calculated using one-way ANOVA, Tukey’s multiple comparisons test. **h** Schematic diagram of a five-chamber social interaction tank for the visually mediated social preference test (VMSP). **i**–**k** Heatmap of *scgn*^+/+^ zebrafish in the adaption session (**i**), interaction session (**j**), and social novelty session (**k**). **l**–**n** Heatmap of *scgn*^+/−^ zebrafish in the adaption session (**l**), interaction session (**m**), and social novelty session (**n**). **o**–**q** Heatmap of *scgn*^−/−^ zebrafish in the adaption session (**o**), interaction session (**p**), and social novelty session (**q**). **r** Frequency in the interaction sector during the interaction session of *scgn*^+/+^, *scgn*^*+/−*^, and *scgn*^*−/−*^ zebrafish. **p < 0.01, *p < 0.05. *P*-values were calculated using one-way ANOVA, Tukey’s multiple comparisons test. **s** Frequency in the novelty sector during the novelty session of *scgn*^+/+^, *scgn*^*+/−*^, and *scgn*^*−/−*^ zebrafish. ****p < 0.0001, *P*-values were calculated using one-way ANOVA, Tukey’s multiple comparisons test. All experiments were performed in three repetitions

First, the social cohesion between two homogeneous zebrafish was assessed by the shoaling test,^32^ in which 4-month-old zebrafish (*scgn*^+/+^, *scgn*^+/−^, or *scgn*^−/−^ siblings) were placed in a tank with their swimming behaviors recorded and analyzed (Fig. 2a). We found that *scgn*^+/+^ zebrafish typically swam in schools, as characterized by higher frequency for short inter-fish distances. In contrast, *scgn*-deficient genotypes showed preferential long inter-fish distances: 5.006 cm from the *scgn*^−/−^ group and 4.766 cm of the *scgn*^+/−^ group compared to 3.741 cm of the wild-type zebrafish (Fig. 2b). Furthermore, *scgn*^+/+^ zebrafish spent 46.7% of their time swimming within a distance of 3 cm or less, while *scgn*^+/−^ and *scgn*^−/−^ groups swim for only 24.4% and 21.3% of the time, respectively (Fig. 2c).

Next, a two-sector tank divided by a transparent plastic board for visualization was applied for the behavioral study.^32^ A group of two zebrafish was placed on one side, and a single *scgn*^+/+^, *scgn*^+/−^, or *scgn*^−/−^ testing zebrafish was placed on the other side (Fig. 2d). The *scgn*^+/+^ fish displayed a stronger grouping tendency, with markedly longer time in the interactive sector than in the empty sector (Fig. 2e). However, *scgn*^+/−^ or *scgn*^−/−^ zebrafish demonstrated an apparent reduction in social durations and a seven-fold increase in border-crossing frequencies (Fig. 2f, g).

Also, we performed the visually mediated social preference (VMSP) test to detect social preference and social novelty in two consecutive sessions within the same place.^33^ A single *scgn*^+/+^, *scgn*^+/−^, or *scgn*^−/−^ zebrafish testee was placed in the center of a tank with five chambers divided by transparent plastic boards. After an adaption session, the first stranger fish group was placed in the lower left chamber for 5 min (interaction session), followed by a second stranger fish group introduced to the upper right chamber for the next 5 min (novelty session). Behavior recording of the tested individual was conducted during the entire time (Fig. 2h). Relative to *scgn*^+/+^ zebrafish, both *scgn*^+/−^ and *scgn*^−/−^ testees displayed reduced social interaction and social novelty (Fig. 2i–k, l–n, o–q), as assessed by the total time spent in corresponding sectors. Quantitatively, the *scgn*^+/+^, *scgn*^+/−^, and *scgn*^−/−^ testees spent 57.9%, 20.0%, and 28.8% of the time, respectively, establishing social tendencies, and 63.1%, 8.2%, and 6.1%, respectively, interacting with new companions (Fig. 2r, s). Altogether, both *scgn* homozygous or heterozygous deletion could lead to autism-like behaviors in zebrafish, including deficits in both social interaction and social novelty.

### *Scgn* deficiency impairs zebrafish brain development

**Fig. 3:**
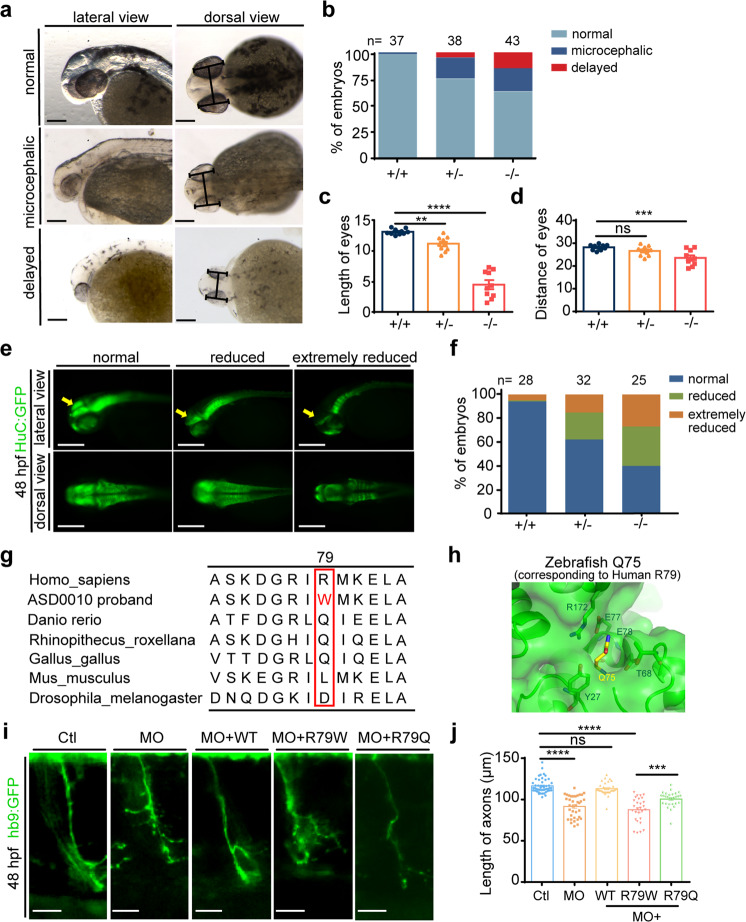
*Scgn* deficiency impairs neuronal development, and R79W mutation is functionally impaired. **a** Bright-field images of zebrafish embryos classified in three groups based on head and body morphology at 48 hpf (hours post fertilization). Microcephalic: the brain size is moderately reduced; delayed: fish development is severely delayed, and the brain size is significantly reduced. Scale bar, 100 μm. **b** Percentage of embryos in each group as defined in A. N stands for the number of embryos examined from three independent experiments. +/+: *scgn*^+/+^; +/−: *scgn*^+/−^; −/−: *scgn*^−/−^. **c** Relative length of eyes of embryos at 48 hpf. Experiments were repeated three times. ****p < 0.0001, **p < 0.01. *P*-values were calculated using one-way ANOVA, Tukey’s multiple comparisons test. **d** Relative distance between a pair of eyes of embryos at 48 hpf. Experiments were repeated three times. ***p < 0.001, ns not significant. *P*-values were calculated using one-way ANOVA, Tukey’s multiple comparisons test. **e** Classification of zebrafish embryos based on the expression level of HuC (elavl3) at 48 hpf. Reduce: the brain size is moderately decreased; Extremely reduced, the brain size is significantly reduced.(Top) Lateral views; (Bottom) dorsal views. **f** Percentage of embryos in each group as defined in E. N stands for the number of embryos used for statistical analysis. All experiments were performed in three repetitions. **g** Multispecies alignment of SCGN protein sequences, Sequence of the ASD0010 proband is included as a comparison. **h** Detailed view of zebrafish SCGN Q75 (yellow stick, equivalent to human R79) and its surrounding residues (PDB:6JLH). Residues located within 4 Å of Q75 are shown in stick representation. **i** Hb9 (green) expression in Tg [Hb9: GFP] transgenic zebrafish. Enlarged views are shown. Ctl: control MO injection; MO: *scgn* MO injection; MO + WT: co-injection of *scgn* MO and wide-type human *scgn* mRNA; MO + R79W: co-injection of *scgn* MO and mRNA encoding SCGN R79W; MO + R79Q: co-injection of *scgn* MO and mRNA encoding SCGN R79Q. All injections were performed at one-cell stage of the Tg [Hb9: GFP]^ml2^ transgenic zebrafish embryos. Scale bar: 20 μm. **j** Statistical results of the length of CaP axons in embryos were treated as in **c**. For each group, ~48 axons from 6 to 12 Tg [Hb9: GFP]^ml2^ transgenic zebrafish embryos are scored. Experiments were repeated three times. ****p < 0.001, ***p < 0.001, ns not significant. *P*-values were calculated using one-way ANOVA, Tukey’s multiple comparisons test. All experiments were performed in three repetitions

As the ASD probands carrying *SCGN* variants displayed delayed overall and neuronal development, we next investigated the roles of *scgn* in zebrafish brain development.^32^ Compared with *scgn*^+/+^ embryos, the size of the head was significantly reduced in *scgn* ^−/−^ embryos at 48 hpf (Fig. 3a, b), consistent with previous reports.^34^ Based on morphology criteria, we classified the embryos into three classes: normal, microcephalic, and severely delayed. Under our experimental conditions, 2% of *scgn*^+/+^ embryos showed abnormal development, whereas 25% of *scgn*^+/−^ embryos and as high as 37% of *scgn*^−/−^ embryos were microcephalic or severely delayed (Fig. 3a, b).

The eye length and the distance between the eyes often reflect the characteristics of brain development in zebrafish.^32^ We found that *scgn*^−/−^ embryos exhibited significantly smaller eyes with shorter distances between the eyes compared with the *scgn*^+/+^ group, and *scgn*^+/−^ embryos showed an intermediate phenotype (Fig. 3c, d). To further characterize the role of Scgn in neuronal development, we used the HuC-GFP transgenic line expressing this early marker of pan-neuronal cells.^35^ Under our experimental conditions, 10% of *scgn*^+/+^ embryos showed extremely reduced midbrain; while ~40% of *scgn*^+/−^ and 60% of *scgn*^−/−^ embryos displayed defects (Fig. 3e, f). Compared with *scgn*^+/+^ embryos, the expression of HuC was substantially reduced in *scgn* mutant embryos, particularly in the midbrain (Supplementary Fig. 2a).

As *scgn* deletion reduced brain size and HuC expression in zebrafish, we next investigated how Scgn might contribute to the zebrafish brain development. The embryo brains were subjected to staining for the cell proliferation marker pHH3 or TUNEL assay for apoptosis.^36^ Relative to the *scgn*^+/+^ group, *scgn*^−/−^ embryos had less than one-third of the pHH3^+^ cells in the lens, midbrain, and diencephalon, suggesting significantly reduced levels of proliferation (Supplementary Fig. 2b, c). Although *scgn* deletion was associated with increased TUNEL puncta in the lens and near the midbrain than those from the wild-type group, such differences were not statistically significant (Supplementary Fig. 2d, e). Therefore, *scgn* might contribute to brain development, mostly via regulating neuronal proliferation in the zebrafish model.

In addition to these observations, we found that *scgn* homozygous or heterozygous deletion led to high mortality rate at 48 hpf, with profound morphological changes, including reductions of the melanin content in the eye, tail bending deficits, developmental delay, and ultimately a shorter life-span (Supplementary Fig. 2f, g). However, over the course of the developmental process, these defects gradually became less noticeable in the surviving zebrafish, likely due to the concurrent development of adaptive changes with better survival chances.

### SCGN R79W is functionally impaired

To further investigate the role of SCGN in regulating neuronal development, we employed the Tg[Hb9:GFP]^ml2^ transgenic line to visualize the motor neurons in zebrafish.^37,38^ Embryos from the *scgn*^−/−^ group displayed abnormal CaP motor neuron morphology, including shortened axonal length and increasing branching, similar to our previous findings.^25^ The *scgn*^+/−^ embryos showed an intermediate phenotype relative to the *scgn*^−/−^ and *scgn*^+/+^ groups (Supplementary Fig. 3a). Specifically, the average length of the CaP axon in *scgn*^−/−^ and *scgn*^+/−^ was 72% and 83% of that in *scgn*^+/+^ zebrafish, respectively (Supplementary Fig. 3b). The average number of branches were 5, 3, and 1 in *scgn*^+/+^, *scgn*^+/−^, and *scgn*^−/−^ zebrafish, respectively (Supplementary Fig. 3c). Thus, the Tg[Hb9:GFP]^ml2^ transgenic line could serve as a suitable model to evaluate *scgn* variants and their potential functional impact.

The *SCGN* gene encodes a calcium-sensing protein with 6 EF-hand domains.^20^ R79 in human SCGN locates in the second EF-hand of the protein. Interestingly, R79 is not a conserved site within SCGN, and it is substituted for Glutamine in zebrafish or Leucine in mice (Fig. 3g). To gain insight into how the R79W substitution might affect SCGN function, we examined the crystal structure of zebrafish Scgn binding to a SNAP-25 peptide. Q75, corresponding to R79 in humans, is located within a pocket predominately formed by charged or polar residues, including Y27, T68, E77, E78, and R172 (Fig. 3h). The substitution of Glutamine or Arginine by Tryptophan in this position could disrupt the local structure due to its large size, and/or diminish the polar interactions.

Next, we used the Tg[Hb9:GFP]^ml2^ transgenic line to evaluate the role of R79W discovered in ASD0010 proband. Knockdown of zebrafish Scgn using a translation blocking morpholino (MO) resulted in abnormally short and branched axons at 48 hpf, functionally similar to Scgn deletion. Importantly, co-injection of mRNA encoding human wild-type SCGN could effectively restore the axonal phenotypes, including the axonal lengths and branch numbers. Meanwhile, SCGN R79Q, capturing the zebrafish Scgn residue at this site, was also able to rescue the axonal morphologies, but the mutation R79W seen in our proband failed to rescue neuronal morphology (Fig. 3i, j). The average axonal length in the MO + R79W group (0.0995 mm) was similar to that of the MO group (0.0985 mm) and was substantially shorter than those in the MO + WT (0.1158 mm) or the MO + R79Q (0.1053 mm) embryos (Fig. 3j). These data corresponded to findings from our structural analysis, suggesting that the R79W substitution would disrupt the normal function of SCGN. Thus, we concluded the p.R79W variant discovered in the ASD patient represents a loss-of-function mutation of *SCGN*.

### SCGN deficiency alters immune response and disrupts the oxytocin signaling pathway

**Fig. 4:**
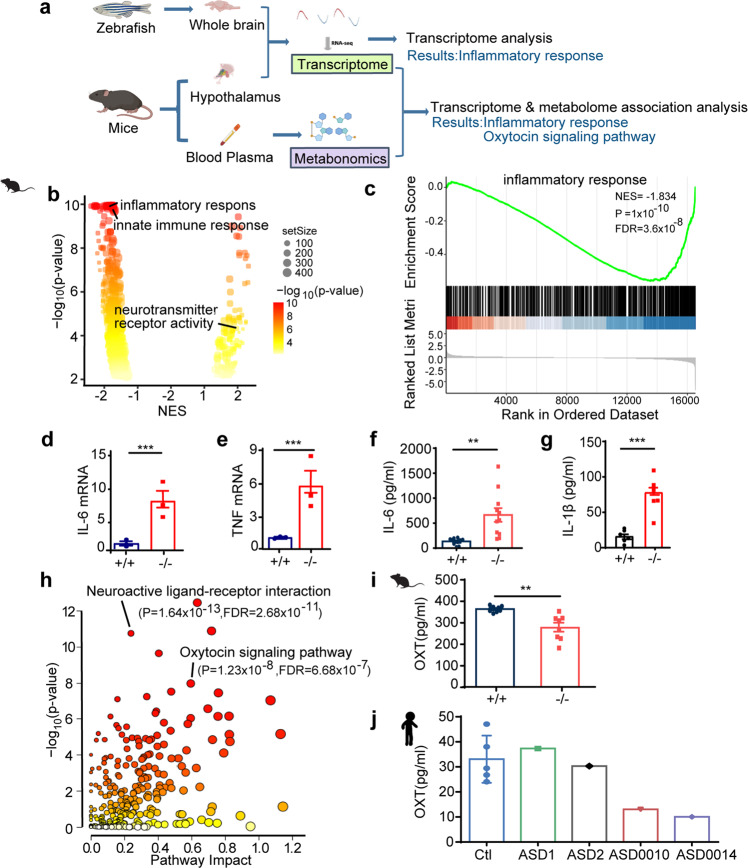
Integrated omics identify SCGN as a key regulator of immune response and oxytocin signaling pathways. **a** Multi-omics profiling of zebrafish and mice samples. Zebrafish whole brain and Mice hypothalamus were extracted for transcriptome analysis. Mice serum was collected for metabolome analysis. Transcriptome and metabolome from mice were further integrated for association analysis. **b** Volcano plots of GSEA results based on differentially expressed genes in the hypothalamus of *scgn*^+/+^ and *scgn*^−/−^ mice. The dot color indicated the −log_10_ *p*-value in each pathway, while the dot size represented the gene number of each pathway. NES Normalized Enrichment Score. Volcano plots shows inflammatory and immune response pathways were signatures based on mice transcriptome analysis. **c** GSEA plot shows the signatures of the immune response in hypothalamu transcriptomes by *scgn*^−/−^ mice compared to *scgn*^+/+^ mice. NES Normalized Enrichment Score. *P,* *p*-value. FDR False Discovery Rate. **d**, **e** Relative mRNA expression of immune and inflammation related genes upon brain tissue in *scgn*^+/+^ and *scgn*^−/−^ mice after exposure to Bleed challenges (*n* = 3). **d** IL-6; **e** TNF. *N* = 3 for each group. Data were presented as mean ± SD, *P*-values were calculated using one-way ANOVA, Tukey’s multiple comparisons test. ***p < 0.001, **p < 0.01, *p < 0.05, ns not significant. **f** Plasma IL-6 protein level is increased in *scgn*^−/−^ mice relative to control. **p < 0.01. Differences among groups by unpaired Student *t*-test. Experiments above were repeated three times. **g** Plasma IL-1β protein level is increased in *scgn*^−/−^mice. ***p < 0.001. *P*-values were calculated using unpaired Student *t*-test. Experiments above were repeated three times. **h** Mice transcriptome& metabolome association analysis reveals difference of oxytocin signaling pathways and neuroactive ligand-receptor interaction. **i** Plasma oxytocin concentrations of *scgn*^−/−^ mice is decreased. *N* = 8 for each group. Data were presented as mean ± SD, *P*-values were calculated using one-way ANOVA, Tukey’s multiple comparisons test. **p < 0.01. **j** Plasma concentrations of oxytocin in 3 control individuals, 2 ASD children without *scgn* mutation, ASD0010 and ASD0014 proband. ASD0010 and ASD0014 showed a lower plasma oxytocin levels than control and ASD1, ASD2. Ctl: control, healthy children; ASD1 and ASD2: ASD probands who do not carry *scgn* mutations; ASD0010: ASD individual carries mutation SCGN p.R79W; ASD0014: ASD individual with mutation *scgn* c.702 + 2 T > G. All individual in this test ages are around 3 years old. Experiments above were repeated three times

To more precisely determine how SCGN deficiency contributed to ASD, we used RNA-seq to compare the gene expression profiles from the brains of 2-month-old zebrafish and from the hypothalamus of mice, focusing on differences between control (*scgn*^+/+^) and deficient (*scgn*^−/−^) animals (Fig. 4a). In parallel, as the high expression of *Scgn* in mice hypothalamus was associated with functional regulation of hormone secretion,^29^ we analyzed the serum metabolome differences between *Scgn*^+/+^ and *Scgn*^−/−^ mice (Fig. 4a). Collectively, these omics data suggested that SCGN deficiency affected similar pathways in zebrafish and mice. Remarkably, profiles from both animal models exhibited comparable alterations in signaling activities found in ASD probands with SCGN mutations.

Specifically, principal component analysis (PCA) of transcriptome analysis in mouse brain based on rlog values of all expressed genes showed that gene expression in Scgn-deficient animals was substantially different than in control mice (Supplementary Fig. 4a). Gene Set Enrichment Analysis (GSEA) indicated that the differentially expressed genes (DEGs) that distinguished the two groups were enriched in pathways such as neurotransmitter receptor activity, consistent with known functions of SCGN (Fig. 4b). Interestingly, multiple immune regulatory pathways were also enriched, including inflammatory and innate immune response signals (Fig. 4b, c, Supplementary Fig. 4c). To further confirm our observations, we performed quantitative RT-PCR and compared the expression of multiple genes responsible for immune response in the *Scgn* mouse models. SCGN deficiency led to the up-regulation of critical pro-inflammatory genes, including IL-6 and TNF (Fig. 4d, e). The elevation of IL-6 and IL-1β protein levels in *Scgn*^−/−^ mice was further confirmed by ELISA (Fig. 4f, g). Altogether, our results indicated that SCGN deficiency could contribute to abnormal inflammation.

As SCGN typically regulated hormone secretion, we compared the serum metabolome between *Scgn*^+/+^ and *Scgn*^−/−^ mice. Results from integrated metabolomics and transcriptome analyses revealed that SCGN deficiency led to disrupted oxytocin signaling and the neuroactive ligand-receptor interaction pathway (Fig. 4h). Specifically, deletion of SCGN affected the expression of many genes in the oxytocin pathway, such as *Oxt*, *Ppp3cb*, and *Plcb3* (Supplementary Fig. 4d). Oxytocin is a peptide hormone produced in the hypothalamus and released by the posterior pituitary. ELISA results confirmed that the serum oxytocin levels decreased by approximately 30% in *Scgn*^−/−^ mice, relative to aged-matched control animals (Fig. 4i). Finally, we examined the serum levels of oxytocin in children diagnosed with ASD and age-matched healthy volunteers. Similar to what has been reported,^39^ serum oxytocin levels in healthy children were in a range of 24–47 pg/ml. Comparatively, both ASD0010 and ASD0014 exhibited reduced amounts of oxytocin in their blood, 13 and 19 pg/ml, respectively. To be noted, the levels of oxytocin were found to be normal in the serum of three ASD children with wild-type *SCGN* gene (Fig. 4j). Our data demonstrate that the amount of oxytocin may vary among ASD patients, while functional mutations in *SCGN* gene may negatively affect serum oxytocin levels, emphasizing that unique pathways can lead to ASD and the importance of considering personalized treatment for ASD.

### SCGN deficiency impairs immune responses and oxytocin secretion in zebrafish

**Fig. 5:**
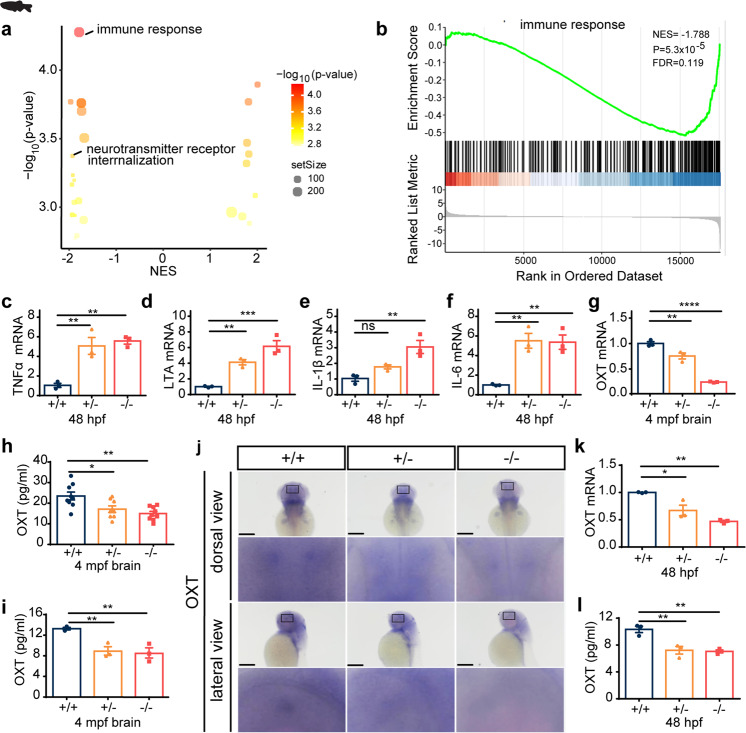
*Scgn* deficiency impairs immune response and decrease oxytocin secretion in zebrafish. **a** Volcano plots of GSEA results based on differentially expressed genes in the brains of 4 month old of *scgn*^+/+^ and *scgn*^−/−^ zebrafish. The dot color indicated the −log_10_ *p*-value in each pathway, while the dot size represented the gene number of each pathway. **b** GSEA plot showed the signatures of the immune response in transcriptomes in *scgn*^+/+^ and *scgn*^−/−^ zebrafish brains. NES Normalized Enrichment Score. *P* *p*-value. FDR False Discovery Rate. **c**–**f** Relative mRNA expression of immune and inflammation related genes upon *scgn*^+/+^ and *scgn*^−/−^ zebrafish. **c** TNFα; **d** LTA; **e** IL-1β; **f** IL-6. *N* = 3 for each group. +/+: *scgn*^+/+^, +/−: *scgn*^+/−^, −/−: *scgn*^−/−^. Data were presented as mean ± SD, *P*-values were calculated using one-way ANOVA, Tukey’s multiple comparisons test. ***p < 0.001, **p < 0.01, *p < 0.05, ns not significant. **g** Relative mRNA expression of oxytocin in adult fish brain. *N* = 3 for each group. Data were presented as mean ± SD, *P*-values were calculated using one-way ANOVA, Tukey’s multiple comparisons test. ****p < 0.0001, **p < 0.01. **h** *Scgn*^−/−^ and *scgn*^+/−^ had lower brain oxytocin concentrations than *scgn*^+/+^. +/+: *scgn*^+/+^, +/−: *scgn*^+/−^, −/−: *scgn*^−/−^. *N* = 3 for each group. Data were presented as mean ± SD, *P-*values were calculated using one-way ANOVA, Tukey’s multiple comparisons test. **p < 0.01, *p < 0.05. **g** Relative mRNA expression of oxytocin in adult fish brain. *N* = 3 for each group. Data were presented as mean ± SD, *P-*values were calculated using one-way ANOVA, Tukey’s multiple comparisons test. ****p < 0.0001, **p < 0.01. **i** *Scgn*^−/−^ and *scgn*^+/−^ had lower plasma oxytocin concentrations than *scgn*^+/+^. Data were presented as mean ± SD, *P*-values were calculated using one-way ANOVA, Tukey’s multiple comparisons test. **p < 0.01. *N* = 3. **j** Whole-mount in situ hybridization in dorsal view (Top) and lateral view (Down) of oxytocin in 48 hpf zebrafish embryos upon *scgn*^+/+^, *scgn*^+/−^, and *scgn*^−/−^. The black rectangles label the position of oxytocin^+^ cells. Scale bar: 120 μm. **k** QRT-PCR analysis of the relative transcription level of oxytocin at 48 hpf upon 3 groups. *N* = 3 for each group. Data were presented as mean ± SD, *P-*values were calculated using one-way ANOVA, Tukey’s multiple comparisons test. **p < 0.01, *p < 0.05. **l** Oxytocin concentration levels in 48 hpf upon *scgn*^+/+^, *scgn*^+/−^, and *scgn*^−/−^. Data were presented as mean ± SD, *P-*values were calculated using one-way ANOVA, Tukey’s multiple comparisons test. **p < 0.01

When we compared the gene expression profiles of *scgn*^+/+^ and *scgn*^−/−^ zebrafish, we made consistent observations of those made in mice. The DEGs were highly enriched for immune response pathways (Fig. 5a, b and Supplementary Fig. 4e, f). By quantitative RT-PCR, we confirmed that multiple pro-inflammatory genes, including TNFα, LTA, IL-6, and IL-1β, were all significantly up-regulated in *scgn*^−/−^ zebrafish (Fig. 5c–f). Also, both the oxytocin mRNA and proteins levels were significantly lower in the brains of 4 mpf *scgn*^+/−^ and *scgn*^−/−^ zebrafish, relative to the age-matched *scgn*^+/+^ group (Fig. 5g, h). Furthermore, we found that *scgn* ^+/−^ and *scgn*^−/−^ zebrafish had ~31% reduction in the serum oxytocin levels relative to *scgn*^+/+^ controls (Fig. 5i).

To determine whether SCGN affected the oxytocin levels during the embryonic stage, we examined the mRNA and protein expressions of oxytocin at 48 hpf. In situ hybridization (ISH) results indicated that oxytocin was expressed near the lateral edge of the neural tube in zebrafish embryos (Fig. 5j). Both ISH and quantitative RT-PCR analysis revealed a dramatic reduction in oxytocin mRNA levels in *scgn*^+/−^ and *scgn*^−/−^ zebrafish compared to the *scgn*^+/+^ group (Fig. 5k). Oxytocin protein levels were also decreased in the *scgn*^+/−^ and *scgn*^−/−^ zebrafish compared to control embryos (Fig. 5l). Zebrafish has two oxytocin receptors, *OXTR1* and *OXTR2*. Interestingly, we found that both *scgn*^+/−^ and *scgn*^*−/−*^ zebrafish displayed increased expression of *OXTR1*, while only *scgn* homozygous deletion elevated the level of *OXTR2* (Supplementary Fig. 5a, b). We speculated that the observed up-regulation of oxytocin receptors could be a feedback response to the decreased oxytocin level resulting from *scgn* deficiency. Altogether, these data demonstrate that functional loss of SCGN leads to decreased oxytocin in zebrafish and mice, similar to that seen in ASD probands with *SCGN* mutations.

### Administration of oxytocin analogue, dexamethasone, and aspirin antagonizes *scgn* deficiency in zebrafish

**Fig. 6:**
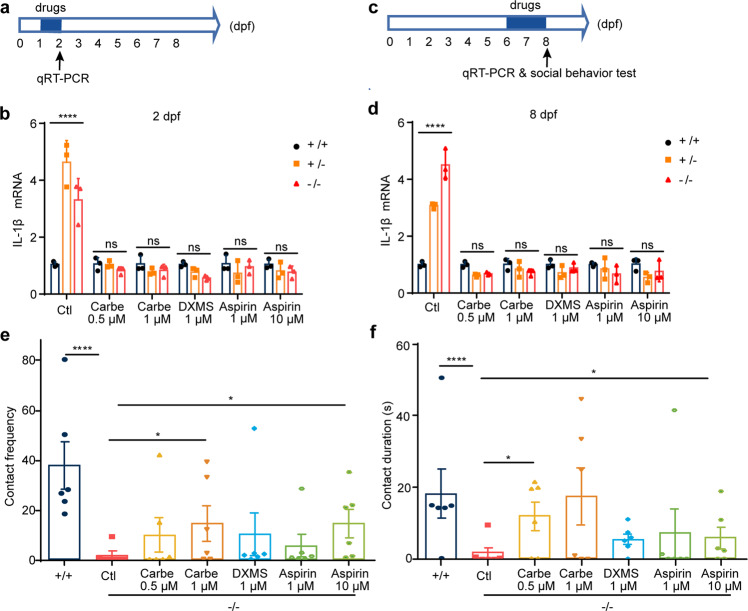
Administration of oxytocin analogue, dexamethasone and aspirin restores immune response and social behavior in zebrafish. **a** Experimental set-up and drugs exposure to 1-day-old embryos. Zebrafish larvea are performed with different concentration drugs (carbetocin, dexamethasone, or aspirin), respectively. qRT-PCR analysis of the relative transcription level of IL-1β at 2 dpf upon three groups. **b** Carbetocin, DXMS, and aspirin markedly decreases expression of pro-inflammatory cytokines IL-1β, Ctl, egg-water administration, Carbe 0.5 μM, Carbetocin 0.5 μM treatment; Carbe 1 μM, Carbetocin 1 μM treatment; DXMS 1 μM, dexamethasone 1 μM treatment; Aspirin 1 μM, Aspirin 1 μM treatment; Aspirin 10 μM, Aspirin 10 μM treatment. +/+: *scgn*^+/+^, +/−: *scgn*^+/−^, −/−: *scgn*^−/−^. DXMS Dexamethasone, a synthetic corticosteroid used to treat inflammation, Aspirin also known as acetylsalicylic acid. *P-*values were calculated using one-way ANOVA, Tukey’s multiple comparisons test. ****p < 0.0001. Ns, not significant. **c** Six-day-old embryos were exposed to drugs for 2 days, respectively. IL-6 level and social behavior tests were performed on 8 dpf. **d** Carbetocin, DXMS and aspirin markedly decreases expression of IL-1β. *P-*values were calculated using one-way ANOVA, Tukey’s multiple comparisons test. ****p < 0.0001. Ns not significant. **e** Contact frequency in larvea are rescued with Carbetocin and aspirin administration. +/+: *scgn*^+/+^, −/−: *scgn*^−/−^. *P-*values were calculated using one-way ANOVA, Tukey’s multiple comparisons test. ****p < 0.0001. *p < 0.05. **f** Duration of larvae body contact can be rescued with Carbetocin and aspirin administration. +/+: *scgn*^+/+^, −/−: *scgn*^−/−^. *P*-values were calculated using one-way ANOVA, Tukey’s multiple comparisons test. ****p < 0.0001.*p < 0.05

Thus far, our studies had established that SCGN deficiency results in defects in immune response and oxytocin production. Next, we explored whether anti-inflammation drugs and oxytocin supplementation could rescue *scgn* deficiency, particularly the neuronal developmental impediment noted in zebrafish. We chose two anti-inflammatory drugs, dexamethasone (DXMS), a synthetic corticosteroid, and aspirin (acetylsalicylic acid). We also tested carbetocin, a synthetic analog of oxytocin with an increased half-life. Zebrafish embryos were exposed to water with or without three different drugs from 1 dpf for 1 day (Fig. 6a). Homozygous or heterozygous deletion of *scgn* again led to a significant increase of the pro-inflammatory gene encoding the cytokine IL-1β (Fig. 6b). One-day treatment with 0.5 or 1 μM carbetocin markedly attenuated the increased expression of IL-1β, resulting in similar expression to that seen in non-treated controls (Fig. 6b). Similarly, DXMS and aspirin could also strongly reduce IL-1β expression in *scgn*^+/−^ and *scgn*^−/−^ zebrafish (Fig. 6b). Thus, carbetocin and anti-inflammatory drugs exhibited protective effects that prevented pro-inflammatory gene expression caused by SCGN deficiency.

Next, we used elder larvae, which displayed more robust social interactions, to examine how these drug treatments could affect immune responses and social behaviors. Zebrafish larvae were exposed to the three individual drugs from 6 to 8 dpf (Fig. 6c). The activation of IL-1β resulting from SCGN deficiency was similarly suppressed by the administration of any of these drugs (Fig. 6d). Next, we examined social behavior in 8-day-old larvae subjected to the two-fish contact assay (Fig. 6e, f). Similar to adult zebrafish, *scgn*^−/−^ larvae displayed less social interaction, as assessed by their contact frequency and duration (Fig. 6e, f). Remarkably, all three drugs could improve the abnormal behavior, with the higher doses of carbetocin and aspirin displaying the most dramatic improvement (Fig. 6e, f). Administration of carbetocin (1 μM) restored nearly 2/3 of *scgn*^−/−^ zebrafish to the level of the *scgn*^+/+^ control, as determined by both contact frequency and cumulative contact time. On the other hand, administration of aspirin (10 μM) rescued the social interaction for 1/3 to 1/2 of *scgn*^−/−^ zebrafish. Consistently, aspirin administration significantly reduced the expression of IL-6, IL-10, and IL-12 in *scgn*^−/−^ zebrafish (Supplementary Fig. 6).

### Oxytocin administration improves impaired social novelty and repetitive behaviors in SCGN-deficient mice

Next, we explored whether the administration of oxytocin could rescue abnormal behaviors of SCGN-deficient mice. The paraventricular nucleus (PVN) in the hypothalamus was the focus of our analysis because of the high levels of SCGN expression and oxytocin production in this region.^23,40^ Using the AAV technology, we were able to deplete the majority of *Scgn* expression in the PVN (shScgn and shControl) (Supplementary Fig. 7a, b). Consistent with our data (Supplementary Figs. 4d, 7c), depletion of *Scgn* in the PVN led to a reduction of more than half of the plasma oxytocin levels. As a comparison, depletion of *Scgn* did not alter the plasma levels of norepinephrine and thyroxine, two other hormones, suggesting that *Scgn* specifically regulates the secretion of certain hormones, such as oxytocin (Supplementary Fig. 8a). Furthermore, we detected a significant increase of activated microglia in SCGN-deficiency mice, consistent with the notion that microglia are key mediators of neuro-inflammatory processes.^41^ (Supplementary Fig. 8b, c).

**Fig. 7:**
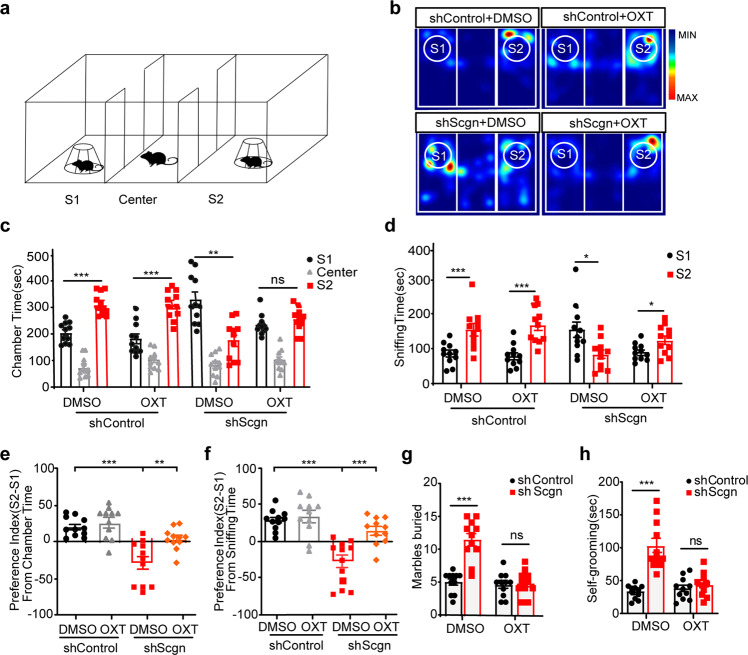
Oxytocin treatment increases social novelty and decreases repetitive behaviors in SCGN-deficiency mice. **a** Schematic of social novelty recognition (three-chamber) test. Time spent interacting with S1(a familiar mouse) or S2 (a novel mouse) is shown for each genotype when injected with Control-shRNA EGFP and Scgn-shRNA EGFP AAV virus in PVN. **b** Representative heatmaps showing the duration and location of the shControl and shScgn mice during the 10 min social novelty recognition test. Warmer colors (red) indicate a greater amount of time spent exploring by the mice. shControl: PVN Control-shRNA injection mouse. shScgn: PVN Scgn-shRNA injection mouse. DMSO: 0.1% DMSO administration. OXT: 1 mg/kg Oxytocin administration. S1: a familiar mouse. S2: a novel mouse. **c** Bar chart showing chamber times spending interacted with S1 or a novel mouse S2 of shControl and shScgn mouse. S1: a familiar mouse. center: center region in chamber. S2: a novel mouse. shScgn: PVN Scgn-shRNA injection mouse. DMSO: 0.1% DMSO administration. OXT: 1 mg/kg Oxytocin administration. S1: a familiar mouse. S2: a novel mouse. *P*-values were calculated using one-way ANOVA, Tukey’s multiple comparisons test. ***p < 0.001, *p < 0.05. ns not significant. **d** Bar chart showing sniffing times spending with S1 or S2 of shControl and shScgn mouse. DMSO: 0.1% DMSO-treated. OXT: 1 mg/kg Oxytocin-treated. *P*-values were calculated using one-way ANOVA, Tukey’s multiple comparisons test. ***p < 0.001, *p < 0.05. **e** Statistics of the social preference index (S2-S1/total) from chamber time in **c**. *P*-values were calculated using *t*-test. ***p < 0.001, **p < 0.01. (**f**) Statistics of the social preference index (S2-S1/total) from sniffing time in **d**. *P*-values were calculated using *t*-test. ***p < 0.001. **g** Statistics of marbles burying numbers in shControl and shScgn mice treated with DMSO or OXT. The data are presented as the mean ± SD. *n* = 12. ***p < 0.001. ns not significant. **h** Statistics of self-grooming times in shControl and shScgn mice treated with DMSO or OXT. The data were presented as the mean ± SD. *n* = 12. ***p < 0.001. ns not significant

Next, we performed a series of experiments to compare the behavior of shScgn and shControl mice.^42^ Social approach was analyzed first, using a three-chamber test (Supplementary Fig. 7d, e). In this assay, a novel mouse (S1) was placed in the left chamber, and a novel object (O) was placed in the right chamber. Both shScgn and shControl mice spent more time in the S1 chamber than in O chamber, as determined by chamber times (Supplementary Fig. 7e, f, shControl: *p* < 0.0001; shScgn: *p* < 0.05) and sniffing times (Supplementary Fig. 7g, shControl: *p* < 0.0001; shScgn: *p* = 0.08). No significant differences were detected between shScgn and shControl mice in terms of social preference of S1 or O, calculated by chamber times (Supplementary Fig. 7h) and sniffing times (Supplementary Fig. 7i). Secondly, we performed the social novelty recognition test, in which the familiar mouse (S1) was placed in the left chamber with a novel mouse (S2) placed in the right chamber (Fig. 7a). ShControl mice spent ~52% of their time exploring the S2 stranger; in contrast, shScgn mice spent only about 30% of the time with S2, suggesting that depletion of *Scgn* in the PVN could induce ASD-like behaviors (Fig. 7b, c). After administration of oxytocin, shScgn mice were found to spend more time with S2 (Fig. 7b, c). Similarly, oxytocin administration also significantly increased the sniffing time of the shControl mice with S2 (Fig. 7d). As a consequence, the social preference index (Fig. 7e, *p* < 0.01) and sniffing time (Fig. 7f; *p* < 0.001) were both profoundly increased by oxytocin administration. Thus, treatment with oxytocin could improve the impaired social novelty behaviors of *Scgn*-deficient mice.

Lastly, we performed marble burying and self-grooming tests for shControl and shScgn mice. shScgn mice showed a remarkable increase in the number of embedded marbles, similar to the whole-body *Scgn* knockout mice. Quantitatively, shControl and shScgn mice buried an average of 5 and 11 marbles, respectively (Fig. 7g, *p* < 0.01). Also, shScgn mice spent more time self-grooming than the control mice, as shown by an average of 100 s for shScgn mice and 40 s for shControl mice (Fig. 7h). Impressively, oxytocin treatment in shScgn mice led to behaviors similar to shControl mice in both the marbles burying and self-grooming tests (Fig. 7g, h). Therefore, the PVN is a key region in the brain for the functional execution of *Scgn*. Depletion of *Scgn* in the PVN can result in ASD-like behaviors, which can be corrected by oxytocin treatment.

### The SCGN network links the oxytocin pathway and immune responses in ASD

**Fig. 8:**
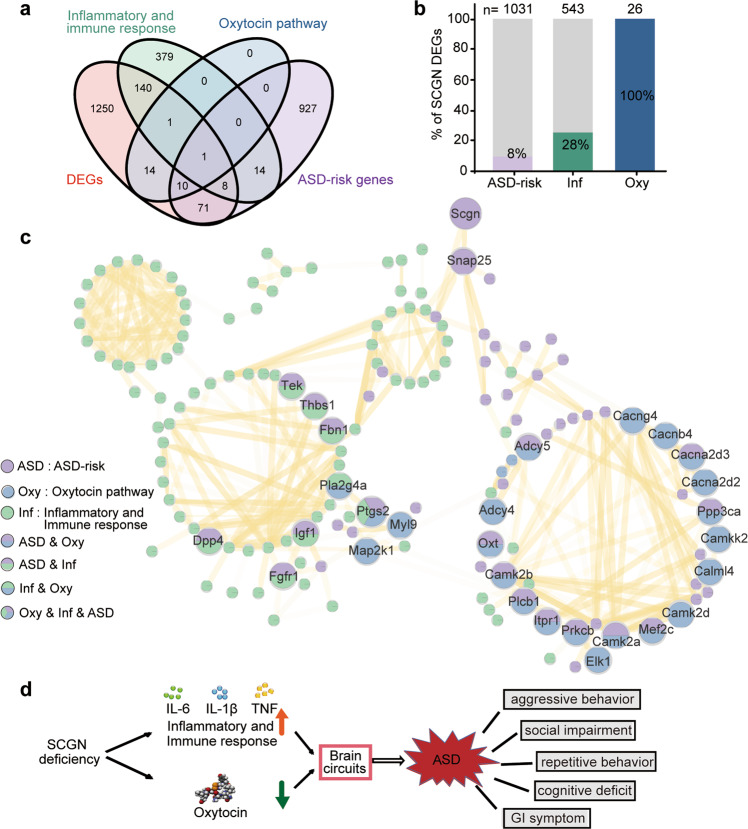
SCGN connects oxytocin and immune response pathways with ASD. **a** Venn-diagram showing the overlap genes between four different gene sets: DEGs: the differentially expressed genes detected in *Scgn*^−/−^ mice RNA-seq. Inflammatory and immune response: the genes enriched in inflammatory and immune response by GSEA. Oxytocin pathway: the genes enriched in oxytocin pathway. ASD-risk genes: the genes related with ASD based on SFARI. **b** The number and ratio of SCGN DEGs associated with ASD, immune response, and oxytocin signaling pathway. 1495 genes that are differentially expressed in the brains of *Scgn*^+/+^ and *Scgn*^−/−^ mice are used for analysis. ASD-risk ASD-risk genes from the SFARI database (March 2022). Inf inflammatory and immune response, Oxy oxytocin pathway. N stands for the number of genes in the KEGG database. **c** The network representing protein interaction relationship between DEGs in ASD-risk, oxytocin pathway. inflammatory and immune response based on *scgn*^−/−^ mice RNA-seq. The interaction confidence was filtered by 0.7. The line transparency indicated the degree of confidence. Purple dots represent ASD-risk genes, blue represent genes associated with the oxytocin pathway, green represent genes associated with inflammatory and immune responses, and the multicolor dots indicate overlap of function and pathway. **d** SCGN deficiency impairs oxytocin secretion and activates the inflammatory pathway, increasing the risk of ASD. Conversely, SCGN deficiency can be treated by administration of oxytocin and/or anti-inflammatory drugs

Given the little available knowledge of the regulatory network for ASD, a better understanding of the interconnection of ASD-associated genes may uncover key features of the heterogeneity of autism in order to develop more effective therapeutic strategies.^43^ By surveying 1495 genes that were differentially expressed in the hypothalamus of *Scgn*^+/+^ and *Scgn*^−/−^ mice, we found that 90 genes were associated with ASD and 150 were related to immune responses (Fig. 8a, b). A total of 26 DEGs were involved in the oxytocin pathway (Fig. 8a, b). Notably, all of these 26 genes were affected by the loss of SCGN, suggesting that the disrupted oxytocin pathway was one of the major causes of *Scgn*-deficiency-associated ASD.

To further illustrate the connection among SCGN-regulated genes, we studied a network of 226 DEGs that were involved in ASD, the oxytocin pathway, and/or the inflammatory pathway, using the STRING database (Fig. 8c). The resulting network suggested that SCGN regulated the oxytocin and the immune response pathways via SNAP-25, whose abnormal expressions and/or mutations have been associated with ASD and other neurological disorders.^16^ We found that 24 genes, including *Camk2b, Adcy5, Cacna2d3, Camk2a, Itpr1, Plcb1, Prkcb, Mef2c*, and *Ptgs2*, were ASD-risk genes that were involved in the oxytocin pathway. Among them, CAMK2A and CAMK2B are Calcium/calmodulin-dependent protein kinase II (CaMKII) isoforms with pivotal roles in neuronal development.^44,45^ The network also illustrated 97 ASD-risk genes that were associated with the inflammatory/immune response pathway, including *Tek, Fbn1, Dpp4, Igf1, Thbs1, Fgfr1*, and *Ptgs2*. Specifically, *Ptgs2* was found to be a common gene shared by the oxytocin pathway and the inflammatory/immune response pathway,^46,47^ which encodes cyclooxygenase-2 (COX-2), a key enzyme in prostaglandin biosynthesis normally induced during inflammation or by oxytocin. This analysis is consistent with our data that aspirin, an inhibitor of COX-2, attenuates social dysfunction in *scgn*^*−/−*^ zebrafish larvae (Fig. 6b–f). Overall, our network further illustrates that SCGN deficiency increases the risk of ASD by disrupting the oxytocin signaling and immune response pathways.

## Discussion

An urgent need in the ASD research is to identify new risk factors to understand the pathogenic mechanisms, which may facilitate the development of personalized therapies for this heterogeneous disease. Here, we identified *SCGN* as a novel ASD-risk gene from a large ASD cohort of 168 cases, with two probands carrying different mutations in SCGN exhibiting identical neural pathological of ASD. Supportively, we showed that heterozygous and homozygous deletion of *scgn* in zebrafish led to ASD-like behaviors and development defects, mimicking ASD mothers carrying SCGN loss-of-function mutations.

Furthermore, we showed that *Scgn* deficiency in both zebrafish and mice led to elevated pro-inflammatory responses and reduced oxytocin levels. Strikingly, the administration of either anti-inflammatory drugs like aspirin, or oxytocin analogs, could attenuate the ASD-like neuropathological symptoms caused by SCGN deficiency. Notably, SCGN deficiency led to deficits in both social interaction and social novelty in zebrafish, while only the social novelty was damaged in the mouse model with their social interaction undisturbed. This was speculated to be the result of model differences, as depletion of *Scgn* was only triggered in the PVN in the mouse model relative to the full-body knockout in zebrafish. Our study provides a probable mechanistic explanation for the development of ASD, with a potential targetable option for ASD patients suffering from SCGN deficiency. Furthermore, we believe that this study provides the rationale for the development of personalized strategies for the treatment of ASD.

In a previous report, a homozygous mutation (R77H) in the *SCGN* gene was shown to be associated with early-onset colitis, and depletion of SCGN in mice impaired hormone secretion and increased inflammation susceptibility.^29^ These findings, in conjunction with our present report, demonstrate that decreased function of SCGN is associated with altered immune regulation in the intestine and in the brain. In particular, the pro-inflammatory cytokines, including IL-6, IL-1β, and TNFα, were detected in the brain tissue and plasma, while expression profiles of anti-inflammatory cytokines, represented by IL-10, showed no marked alterations in the transcriptome sequencing analysis. Particularly, we found that microglia, the essential cellular mediator of neuro-inflammatory processes, was activated in SCGN PVN-conditional knockdown models. In this prospect, we tested the protective efficacy of anti-inflammatory measurements. The fact that administration of aspirin alone could directly reverse the SCGN-deficiency-caused social deficits highlighted the importance of inflammatory responses in ASD, although the specific dissection for the involvement of aspirin regarding its different mechanisms of action and the understanding of how SCGN contributes to immune activation needed to be addressed in the future. In support, as high as 70% of autistic individuals have GI-related problems such as inflammatory bowel disease, reflux, and diarrhea,^48^ suggesting a possible angle to look at the involvement of genetic regulation of inflammation in the investigation of ASD causes.

SCGN is found primarily in pancreatic β-cells, the central nervous system, and enteroendocrine cells.^20^ It has been reported that SCGN binds with SNAP-25 with high affinity and functions together to regulate hormone secretion.^49^ Remarkably, the deregulation of both genes has been linked with ASD.^50–53^ In this study, we further demonstrate that SCGN is critical for the secretion of oxytocin. Previous studies using single-cell sequencing demonstrate that SCGN + neurons express corticotropin-releasing hormone (CRH), but not oxytocin.^23^ Since CRH is a well-known regulator of oxytocin secretion,^54^ we suspect that SCGN controls the secretion of oxytocin, via CRH. Whether SCGN directly regulates the secretion of oxytocin or indirectly impacts its secretion via other hormones or neurotransmitters, still needs to be addressed. Notably, Nlgn3, another synaptic regulator linked to ASD, has been shown to regulate oxytocinergic signaling with the deletion of *Nlgn3*, resulting in reduced levels of oxytocin in mice.^18^

It is noted that abnormal oxytocin signaling and inflammation regulation have been associated with many neurological disorders, in addition to ASD.^55–57^ Especially, an increase in the number of microglia in Scgn-deficient mice shows that neuroinflammation is linked to the oxytocin pathway in PVN. In response to SCGN deficiency, we identified the potential interconnectivity between oxytocin signaling and the inflammatory/immune response pathway. The fact that the administration of oxytocin analogs could attenuate pro-inflammatory gene expression suggests that disrupted oxytocin might function upstream of the impaired inflammatory pathway. This is supported by numerous studies showing that defects in the oxytocin signaling pathway can lead to pro-inflammatory responses.^58–60^ Children with ASD often have lower levels of oxytocin in their serum compared to their healthy peers.^61^ However, it is very controversial whether the administration of oxytocin could benefit autistic children. Several studies have shown that intranasal oxytocin can improve impaired social behavior in autistic children;^62,63^ a recent study, however, found that oxytocin did not confer any significant benefit.^64^ Possible reasons for these inconsistencies might include the lack of differentiation in the original serum oxytocin levels of participants and ineffective enrichment of the administrated oxytocin at the proper functional sites within the brain. In light of this, our study showed that oxytocin administration could improve social behavioral deficit behaviors in both SCGN mutant zebrafish and mice. Meanwhile, the importance of oxytocin was further addressed using the mouse model with a specific deficit in PVN, which primarily accounted for oxytocin production. It is possible that oxytocin administration may confer more benefits for autistic individuals presented with significantly reduced oxytocin levels, like the ASD probands carrying *SCGN* mutations that were studied here. Moreover, our findings suggest that a potential approach may also be the use of commonly prescribed anti-inflammatory medications, such as aspirin, for improving SCGN-deficiency-associated ASD symptoms. This may help to ease the financial burden and the adverse events associated with the current pharmacological use of dopamine-receptor and serotonin-receptor antagonists for ASD patients.

Altogether, we have shown that functional disruption of SCGN results in autistic symptoms via abnormal pro-inflammatory responses and deregulated hormone secretion, suggesting that the administration of oxytocin and anti-inflammatory drugs as potential treatment for individuals with SCGN deficiency. In the era of precision medicine, these mechanistic studies will help to develop personalized treatments and to broaden the currently limited options for ASD therapeutic interventions.

It was noted that the exact SCGN mutations were kept between the maternal parents as their sons, suggesting an incomplete penetrance probably resulted from the maternal-fetal effect rather than gender- or age-specific phenomenon. It seemed that the lack of maternal serum oxytocin during pregnancy served as the fundamental determinant for the neural development deficits of the fetuses, resulting in the collected ASD symptoms found in these probands. Retrospectively, the neural development of these SCGN mutation-carrying mothers were otherwise safe-guarded by the normal levels of oxytocin from the maternal grandmothers of these proband boys, who exhibited wild-type SCGN genotypes. This conjecture cannot be confirmed at present due to the limited pedigree information available and should be further addressed in future studies with larger cohorts.

SCGN is a critical regulator of synaptic transmission.^29,65^ Impaired synaptic function and changes in axonal branching are among the important mechanisms of autism.^66^ We have not shown the data on axonal branching and dendritic spines and only showed the depletion of axonal length and branching in zebrafish.

## Materials and methods

### Ethics approval and consent to participate

This work is approved by Medical Ethics Committee of Beijing Children’s Hospital, Capital Medical University (2018-k-62). Ethics approval was locally obtained for genetic analyses and/or data sharing for additional patients. Genetic analyses and patient inclusion were performed in accordance with the ethical standards of the GCP principles and relevant national laws and regulations. Informed consent for genetic analyses was obtained from all individual participants included in this study or their legal guardians.

### Subject details

The included 168 patients were recruited from psychiatry outpatient clinics of the Beijing Children’s Hospital, Capital Medical University, and were diagnosed with autism by two independent psychiatrists. Enrollment occurred between July 2019 and January 2020. Written informed consent to participate was obtained from the parents or legal guardians of all of the participants (under the age of 10). Patients who had been diagnosed with ASD according to the criteria of DSM-IV. Patients with a definite history of craniocerebral trauma, neurological disease, and severe physical illness were excluded. The behavior and severity of patients were assessed with the Autism Behavior Checklist, Clancy Autism Behavior Scale, and Childhood Autism Rating Scale.

### Zebrafish strains, care and maintenance

All experiments were approved by the Animal Welfare Center of West China Hospital of Sichuan University. Zebrafish (Danio rerio) were kept in accordance with the University of Sichuan institute animal welfare guidelines. The dark and light conditions were set for 10:14 h. Feedings were given twice per day. The following zebrafish strains were used: *scgn* heterozygous (*scgn*^−/−^) and homozygous (*scgn*^−/−^) mutants; AB Wild-type (WT), Tg Hb9:GFP (WT) and Tg Huc:GFP (WT) stains.

### Mouse studies and stereotactic Injection

All animal experiments followed the guidelines of the Institutional Animal Care and Use Committee at Chongqing Medical University. These studies were approved by the Institutional Review Board and Animal Ethics Committee of Chongqing Medical University. The shRNA was applied to knockdown *Scgn*. Designed AAV delivers of shRNA targeting mouse *Scgn* (shRNA-Scgn-gfp, 5’-GCTGAACTGGAAGAGTATACT-3’) and shControl-gfp were obtained from Beijing Syngentech Co., LTD. Mice were separated into shControl and shScgn groups stochastically. Through a one-week adaptive phase, these mice were anesthetized with isoflurane (3.0% for induction and 1.5% for hold) and then fastened to the stereotaxic device (RWD instruments). shControl and shScgn AAVs were injected into the hypothalamus PVN nucleus ambilateral through an automatic microinjection instrument (World Precision Instruments). The coordinates of target area were as following: AP = − 0.94; ML = ± 0.25; DV = − 4.8. The injection was carried out with a speed of 0.1 ul/min, using a Hamilton needle (1 μl× 1012 viral particles per ml). The needle was kept locally for 10 minutes after injection, then retreated slowly. Four weeks post-viral introduction, behavioral tests were performed. The staining of the marker for microglia cell was used Iba1 antibody (ab178846).

### Total RNA isolation and quantitative RT-PCR

The total RNA of zebrafish embryos at 48 hpf or brain tissues was collected for each sample.^67^ The biological materials were grounded, and the debris was removed using centrifugation. RNA was extracted with TRIzol reagent in combination with the RNeasy Mini Kit (Qiagen, Madrid). Total RNA was separated by Plant Mini Kit (FOREGRNE), and then the Prime Script Reverse-transcription PCR kit (TaKaRa DRR014A) and 1 μg of total RNA were used to synthesize cDNA. Two hundred nanograms of resultant cDNA was used as a template for Real-Time PCR (Bio-Rad) with Real Master Mix Kit (Roche).

### Cell death detection

Zebrafish embryos (48 hpf) were collected, and the egg membranes were manually removed and then fixed with 4% PFA at −4 °C. Then, the heads of the embryos were cut off and soaked in the agar-sucrose mixed gel. Samples were fixed in the standard position. Agarose gel-containing samples were cut into cubes with a side length of 0.3 cm post-solidification. The gel cubes were dehydrated with 30% sucrose for 2 days. OCT embedded heads were frozen at −25 °C for 30 min prior to sectioning at 5 μm per slice and stored at −80 °C until use. Intracerebral apoptotic cells were examined by TUNEL assay. Sample slides were fixed with 4% PFA for 30 min, and permeablized by proteinase K and 0.1% Triton X-100. Following the protocols of TUNEL Apoptosis Assay Kit (Servicebio), samples were further balanced and stained with a reaction mixture and DAPI. Then they were rinsed with PBST, and sealed with quench-resistant sealing fluid. Lastly, Images were captured using a confocal microscope and analyzed using Image J software.

### Phospho-histone H3 (pHH3) staining

Anti-pHH3 antibody (Ser10; Millipore) was used to identify proliferating cells. Similar to mentioned above, selected samples were permeabilized using 0.1% triton X-100-PBST, then circled with PAP PEN. After being terminated with buffer (1% goat serum and 3% BSA diluted by 0.2% triton-PBST), samples were incubated at 4 °C with pHH3 (diluted by 1:500). Second antibody and DAPI were incubated after 24 h according to the manufacturer’s protocol. After rinsing and sealing, images were captured and analyzed.

### Whole-mount in situ hybridization

WISH analysis was carried out as previously described.^25,68^ Briefly, embryos at 48 hpf were fixed and permeabilized with proteinase K (10 mg/ml) for 20 min, and then fixed with 4% paraformaldehyde. Samples were then incubated with digoxigenin (DIG)-labeled antisense RNA probes (oxytocin) at 65 °C overnight. After the probes were washed out with formamide and SSCT concentration gradient, embryos were incubated with alkaline phosphatase (AP)-conjugated antidigoxigenin antibody (1:2000) at 4 °C overnight. NBT/ BCIP (Roche) staining was performed according to the manufacturer’s instructions.

### Zebrafish behavioral methods

Behavior was recorded using Ethovision XT13 software and a digital camera (Basler acA640-120gm, Basler Vision Technology). All behavioral experiments were undertaken after 12:00 p.m. The experiments were carried out in a special room where the light and temperature were kept constant. There were no gender differences in behavior in our recording settings. Therefore, we used a mixed population of male and female adult zebrafish (around 4 months of age) in our experiments.

### Shoaling test

Shoaling was recorded in 35 × 25 cm tanks filled with 8 cm depth of water. Adult fish were introduced into the box to acclimate briefly before being recorded for 10 min. We tracked the fish and measured the distances between every two-individual fish (inter-fish distance).

### Social preference and social novelty test

The test tank was subdivided into two average sections (left and right compartments). WT strangers were introduced into the left compartment, and the test fish were introduced into the right side. Then, the VMSP test (Visually Mediated Social Preference Test) was carried out as described in ref. ^33^

### Collections of brain tissues and plasma

After anesthesia, zebrafish were dissected in PBS at 4 °C. Fish brain or blood was collected in EDTA tubes, centrifuged for 10 min at 4 °C, and then the plasma was immediately pipetted into 1 ml tube. Samples were immediately stored at −80 °C until tested for the quantification of the plasma oxytocin concentrations.

### Three-chamber sociability test

The three-chamber sociability test was displayed as a classic model. Briefly, the social interaction test consisted of three 10 min stages. Mice were allowed to randomly accommodate the chamber for 10 min in the first stage. In the second stage, a novel object and an unfamiliar mouse (S1) were deposited in the right and left chambers, respectively. Test mice were set free to visit different chambers for 10 min. In the last stage, the novel object was substituted by another stranger mouse (S2), with the behavior of the experimental mice recorded for 10 min. Applied Noldus Observer software (Ethovision 11.5) was used to evaluate how much time it took in a single chamber of the three-chamber apparatus. Additionally, the social preference standard was determined by the digital divergence among the chambers with sniffing (S1 vs. object or S2 vs. S1) divided by total time.

### Self-grooming test

Test mice were put in a standard and transparent mouse cage independently to assess self-grooming. After a 10 min adaptive phase, the performance of mice was video recorded by a camera for 10 min. An experienced unbiased observer calculated the amount of time each mouse spent in self-grooming.

### Marble burying test

Twenty black glass marbles were set at 4 × 5 on the surface of a 5 cm-deep packing inside a standard mouse cage (27 × 16.5 × 12.5 cm). Then the mice were individually placed into the cage, and the activity was observed for 30 min. Total marbles buried with > 75% in volume during the 30 min were calculated.

### Enzyme-linked immunosorbent assay (ELISA)

Human or mouse serum, or zebrafish brain samples, were mixed with 0.1% trifluoroacetic acid water (TFA-H_2_O) at 1:1 ratio, centrifuged at 4 °C, evaporated at 4 °C, and stored at 20 °C until recombination.^69^ The OXT levels in samples from different species were measured using corresponding ELISA Kits (JL12828, JL12594, and JL22609, Shanghai Jianglai industrial Limited by Share Ltd., China). All tests were repeated for three times. The IL-6 levels in plasma were measured using Elisa Kit (JL20268-96T), and IL-1β in plasma was measured using ELISA Kit (ab197742).

### Pharmacological drugs and treatments

Carbetocin, DXMS, and Aspirin were purchased from TOPSCIENCE CO., Ltd., China. Zebrafish was individually exposed to different concentrations of drugs dissolved in tank water for a corresponding time in a 500 ml beaker. The control fish was individually placed in tank water under similar conditions. Mice were intraperitoneally injected with Oxytocin (OXT, MCE, CAS:6233-83-6), which was diluted to a final concentration of 1 mg/kg in 0.1% DMSO 1 h prior to injection.

### Minigene construction and mutations

The minigene RHCglo-SCGN was constructed as previously reported.^70^ The minigene vesctor (2 μg) was transfected into HEK293T cells using Hieff Trans Liposomal Transfection Reagent (Yeasen, China). Cells were incubated 48 h and then the RNA was purified with the Cell Total RNA Isolation Kit (Foregene, China).

### Splice site strength evaluation

MaxEntScan method with the Maximum Entropy Model was used to assess the splice site strength for both 3’ (http://hollywood.mit.edu/burgelab/maxent/Xmaxentscan_scoreseq_acc.html) and 5’ splice site (http://hollywood.mit.edu/burgelab/maxent/Xmaxentscan_scoreseq.html). The sequence of the 3’ splice sites must be 23 bases long, including 20 bases in the intron and 3 bases in the exon. Similarly, the sequence of the 5’ splice sites must be 9 bases long, including the 3 bases in the exon and 6 bases in the intron.

### Transcriptome and data analysis

RNA-seq analysis was carried out in accordance with previous studies.^71^ After RNA quantification and qualification, A total amount of 1 μg RNA per sample was used as input material for the RNA sample preparations. Sequencing libraries were generated using NEBNext UltraTM RNA Library Prep Kit for Illumina (NEB, USA) following the manufacturer’s Recommendations. After cluster generation, the library preparations were sequenced on an Illumina Novaseq platform, and 150 bp paired-end reads were generated. Data analysis of transcriptome was performed as previously reported.^71^

### Metabonomics and data analysis

The sample extracts were analyzed using an LC-ESI-MS/MS system (UPLC, ExionLC ADˈ https://sciex.com.cn/; MS, QTRAP System, https://sciex.com/) by Wuhan Metware Biotechnology Co., Ltd. PCA Unsupervised PCA (principal component analysis) was performed by statistics function prcomp within R (www.r-project.org). The data was unit variance scaled before unsupervised PCA. Hierarchical Cluster Analysis and Pearson Correlation Coefficients The HCA (hierarchical cluster analysis) results of samples and metabolites were presented as heatmaps with dendrograms, while pearson correlation coefficients (PCC) between samples were calculated by the cor function in R and presented as only heatmaps. Both HCA and PCC were carried out by R package ComplexHeatmap. For HCA, normalized signal intensities of metabolites (unit variance scaling) are visualized as a color spectrum. Differential metabolites selected Significantly regulated metabolites between groups were determined by VIP ≥ 1 and absolute Log2FC (fold change) ≥ 1. VIP values were extracted from OPLS-DA result, which also contain score plots and permutation plots, was generated using R package MetaboAnalystR.

### Transcriptome and metabonomics joint-pathway analysis

We performed MetaboAnalyst 5.0 for Transcriptome and Metabonomics Joint-pathway Analysis, and we chose the All-pathway enrichment for this analysis.

### Statistics

Unless otherwise specified, statistical analysis was performed by using Student’s *t*-test or Log-rank (Mantel-Cox) test and analyzed using Excel (software, US), ANOVA test using Graphpad Prism 8.0.2 for windows (software, San Diego, California USA, www.graphpad.com). Data are presented as the mean ± SEM or mean ± SD. *P*-value < 0.05 was considered statistically significant.

## Supplementary information

SUPPLEMENTAL Figure

## Data Availability

All RNA-seq raw data of this study are available in GEO (Submission ID: PRJNA877079 and PRJNA87991). The other resource data supporting this paper are presented within the Supplementary Materials. The data that support the findings of this study are available from the authors upon reasonable request.
