# Supplementary material for: SCGN deficiency is a risk factor for autism spectrum disorder
Source: Signal Transduct Target Ther. 2023 Jan 2;8:3. doi: 10.1038/s41392-022-01225-2 (PMC9806109; doi:10.1038/s41392-022-01225-2)
Supplement: Supplementary file 1 — SUPPLEMENTAL Figure [file 41392_2022_1225_MOESM1_ESM.docx]

Supplementary Materials for

*SCGN* deficiency is a risk factor for autism spectrum disorder

Zhe Liu^1,10^, Shuai Tan^2,10^, Lianyu Zhou^3,10^, Li Chen^1^, Mingfeng Liu^1^, Wang Wang^2^ , Yingying Tang^1^,Qin Yang^1^,Sensen Chi^2^, Peiyan Jiang^4^, Yue Zhang^5^, Yonghua Cui^6^, Junhong Qin^1^, Xiao Hu^1^, Shenglong Li^2^, Qi Liu^7^, Lu Chen^1^, Song Li^3^, Ezra Burstein^7^, Wei Li^5*^, Xiaohu Zhang^8*^, Xianming Mo^9*^, Da Jia^1*^

Correspondence to:Da Jia ([JiaDa@scu.edu.cn](mailto:JiaDa@scu.edu.cn))

Xianming Mo ([xmingmo@scu.edu.cn](mailto:xmingmo@scu.edu.cn))

Xiaohu Zhang ([zhangxh_alex@163.com](mailto:zhangxh_alex@163.com))

Wei Li ([liwei@bch.com.cn](mailto:liwei@bch.com.cn))

**This PDF file includes:**

Figures. S1 to S8

Table.S1

**Fig. S1.**


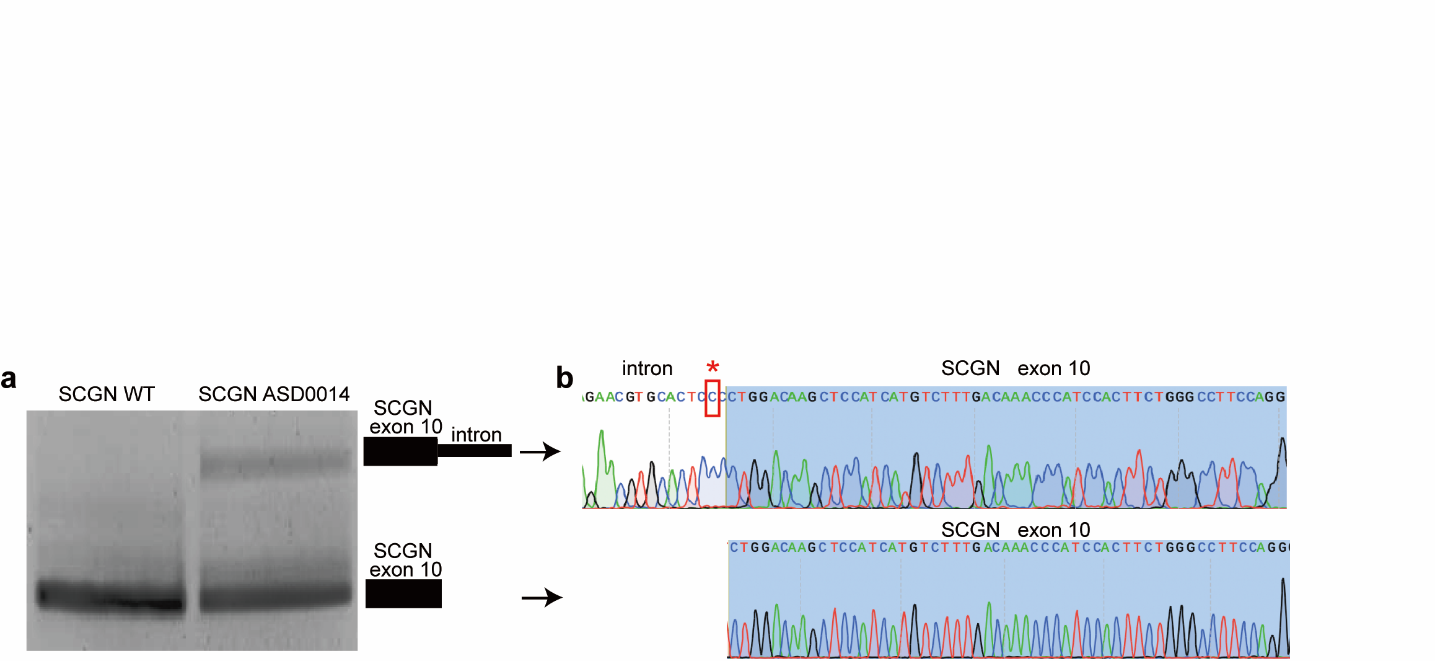


**Fig. S1 ASD0014 proband *SCGN* mutation alters gene splicing.**

1. *SCGN* DNA sequences surrounding ASD0014 variant (NM006998.4:c.702+2T>G) and corresponding WT were cloned into a Minigene reporter vector. The sequences were then transcribed and amplified by RT-PCR.
2. Sanger sequencing results of the top and bottom bands in (a).

**Fig. S2.**


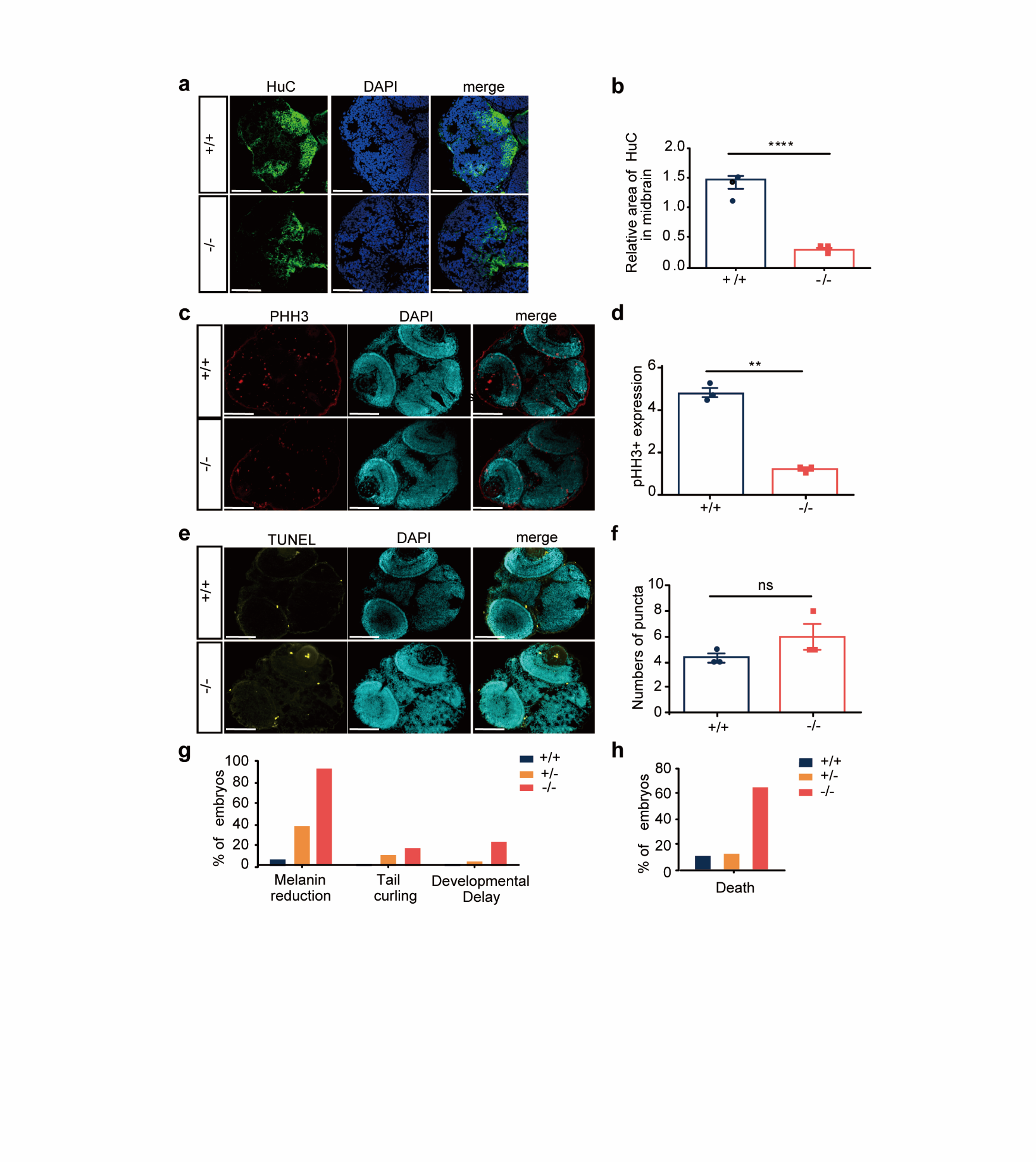


**Fig. S2 Scgn is crucial for zebrafish brain development and morphological characteristics.**

1. Cryostat sections of zebrafish embryos brain at 48 hpf. HuC marked early pan-neuronal cells. DAPI marked nucleus, Scale bar, 40 μm. +/+:*scgn*^+/+^ ,−/−:*scgn*^−/−^ .
2. Statistical results of midbrain HuC area in (a). For each group, 3 embryos are scored. Mean ± S.D. ****P< 0.0001, P values were calculated using T-test.
3. PHH3(red) marked proliferating cell in zebrafish embryos brain, DAPI (turquoise) was used to label nucleus. +/+: *scgn*^+/+^ ,−/−: *scgn*^−/−^ homozygous deletion of the *scgn* gene. Scale bar, 50 μm.
4. Statistical results of the pHH3 puncta in (B). For each group, 3 embryos are scored. PHH3 was used to label proliferating cells. DAPI (turquoise) marked nucleus. Mean ± S.D. **P< 0.01, P values were calculated using T-test.
5. TUNEL(yellow) was used to label apoptosis cell in embryo brain, DAPI (turquoise) marked nucleus. +/+: *scgn*^+/+^,−/−: *scgn*^−/−^ .Scale bar, 50 μm.
6. Statistical results of the apoptosis cell in embryos upon *scgn*^+/+^ and *scgn*^−/−^. Mean ± S.D. ns, none significance. P values were calculated using T-test. ns, not significant. There was not a obviously difference in the number of puncta with TUNEL positive cells per embryo.
7. Abnormal morphological changes in *scgn*^-/-^ and *scgn*^+/-^ embryos at ~ 48 hpf, including severe eye melanin reduction, tail curling and developmental delay, (+/+, n = 45; +/-, n = 47; -/-, n = 42).
8. Abnormal mortality of *scgn*^-/-^ at ~ 2 dpf. All experiments were performed in three repetitions.

**Fig. S3.**


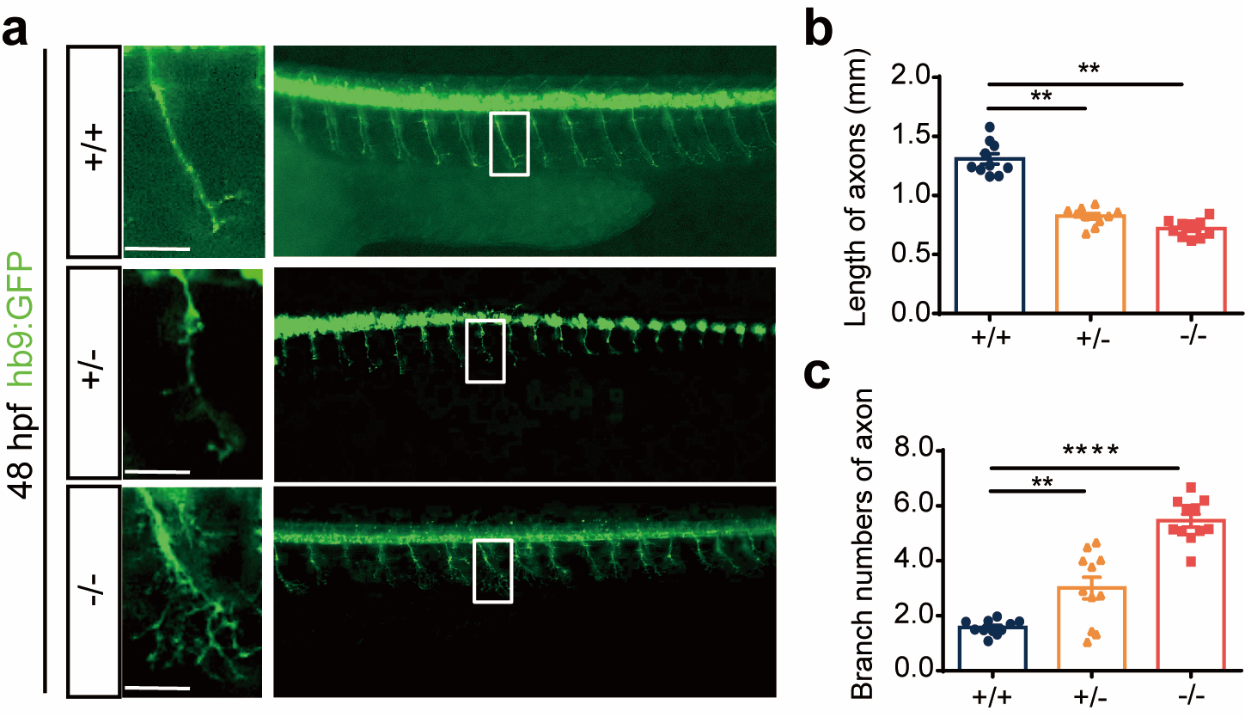


**Fig. S3 Homozygous and heterozygous depletion of Scgn affects axon length and branches in zebrafish.**

1. Morphology of CaP axons from 3 groups zebrafish embryos at 48 hpf. Lateral views and enlarged views are shown. Rectangles in lateral views are shown in the left enlargedly. +/+: *scgn*^+/+^, +/−: *scgn*^+/−^, −/−: *scgn*^−/−^. Scale bar: 100 μm.
2. Statistics of axonal length in 3 different groups at 48 hpf. **p <0.01, *p <0.05. P values were calculated using one-way ANOVA, Tukey's multiple comparisons test.
3. Statistics of axon branch numbers in 3 different groups at 48 hpf, and statistical comparisons were performed using Ordinary one-way ANOVA, ****p < 0.0001, ** p < 0.01. Experiments above were repeated 3 times.

**Fig. S4.**


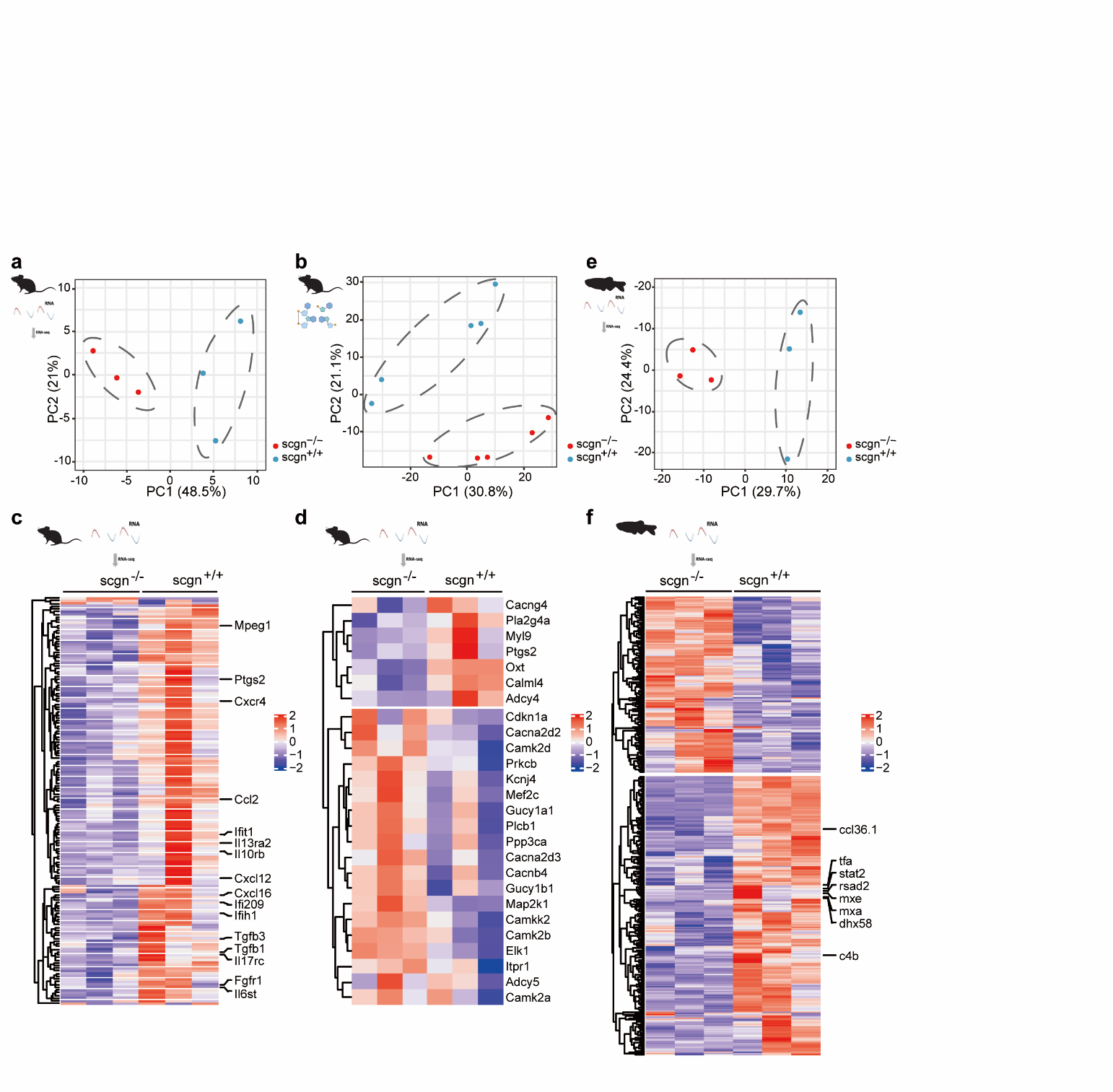


**Fig. S4.**  **Fig. S4 Multi-omics profiling of zebrafish and mice samples.**

(a-b) Principal component analyses (PCA) of all expressed genes or metabolites by mice transcriptome and metabolome analysis, and the number in parentheses indicates the proportion of variance explained by the PCA dimension.

(c-d) Heatmap of the DEGs enriched in the oxytocin and inflammatory-immune response pathway in mice transcriptome. Each row illustrates the expression profile of a single gene and each column represents an experimental sample. Red and blue are indicative of increased and decreased expression, respectively.

(e) PCA of all expressed genes in zebrafish transcriptome analysis, and the number in parentheses indicates the proportion of variance explained by the PCA dimension.

(f) Heatmap of the DEGs in zebrafish transcriptome. Each row illustrates the expression profile of a single gene and each column represents an experimental sample. Red and blue are indicative of increased and decreased expression, respectively. Genes associated with inflammatory and immune response or neural development were marked.

**Fig. S5.**


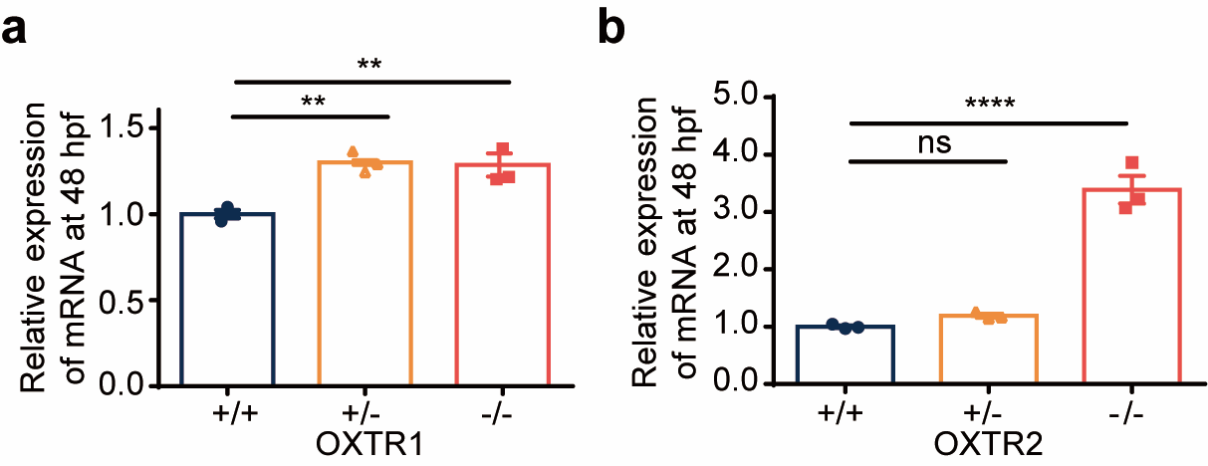


**Fig. S5 Depletion of Scgn increases oxytocin receptor mRNA level in zebrafish embryos.**

1. In 48 hpf, *scgn*^-/-^ and *scgn*^+/-^ had higher mRNA expression OXTR1 (oxytocin receptor1) than *scgn*^+/+^. +/+: *scgn*^+/+^, +/−: *scgn*^+/−^, −/−: *scgn*^-/-^. N = 3 for each group. Data are presented as mean ± SD, P values were calculated using one-way ANOVA, Tukey's multiple comparisons test. **p < 0.01.
2. *Scgn*^-/-^ had higher mRNA expression OXTR2 (oxytocin receptor2) than *scgn*^+/+^ and *scgn*^+/-^. N = 3 for each group. Data are presented as mean ± SD, P values were calculated using one-way ANOVA, Tukey's multiple comparisons test. ****p< 0.0001, ns, not significant. Experiments were repeated 3 times.

**Fig. S6.**


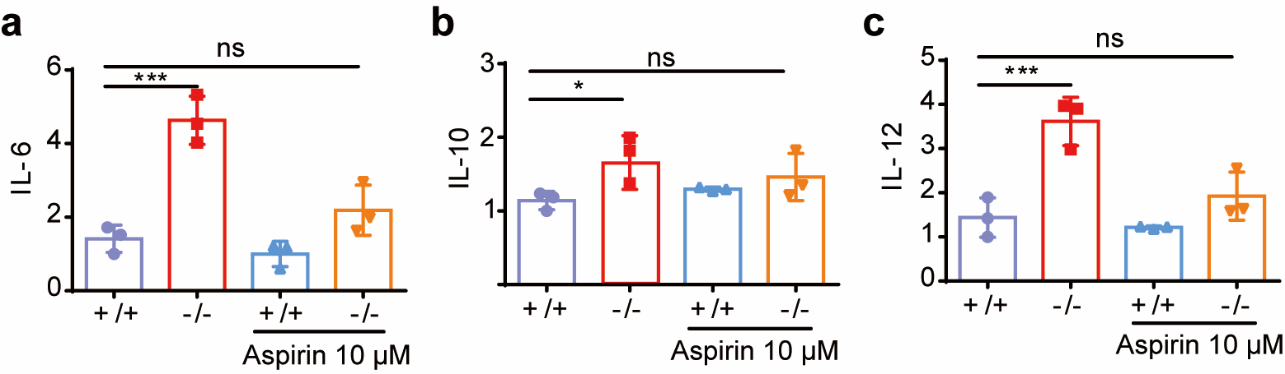


**Fig. S6 Aspirin administration reduces inflammatory response in *scgn*^−/−^** **zebrafish larvea.**

Relative mRNA expression of multiple inflammatory response genes in 8 dpf *scgn*^+/+^ and *scgn*^−/−^ zebrafish, in the presence or absence of aspirin adminstration. (a) IL-6; (b) IL-10; (c) IL-12. *N* = 3 for each group. +/+: *scgn*^+/+^, −/−: *scgn*^−/−^. Data are presented as mean ± SD, P values were calculated using one-way ANOVA, Tukey's multiple comparisons test. ***p < 0.001, Ns, no significant.

**Fig. S7.**


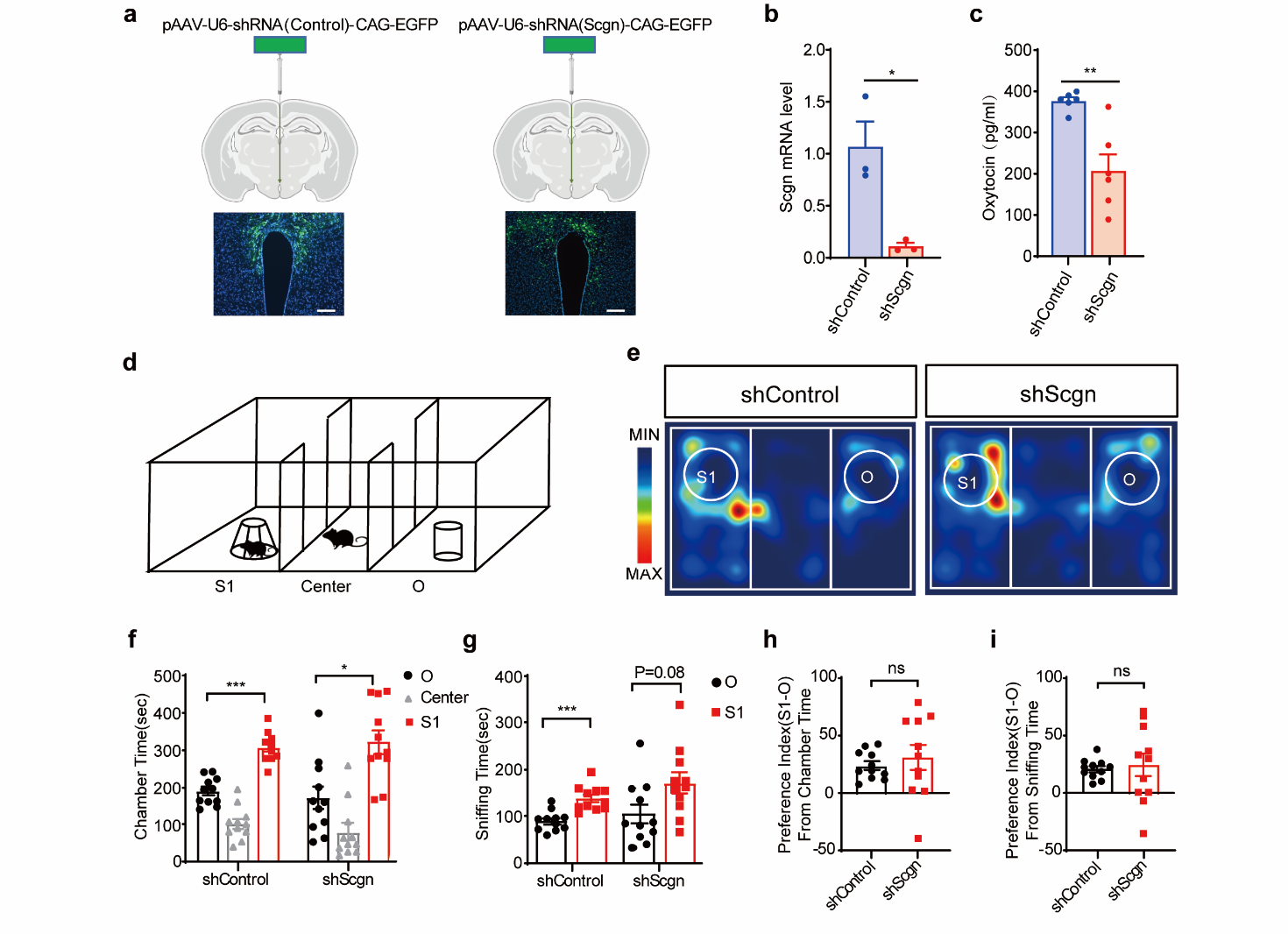


**Fig. S7 Depletion of SCGN in the PVN does not affect social approach of mice.**

1. Schematic representation of Control-shRNA EGFP and Scgn-shRNA EGFP AAV virus injection site with representative images of viral EGFP (n = 20, 5 images per mouse) or Scgn-shRNA EGFP (n = 20, 5 images per mouse) expression in PVN neurons, respectively. Scale bar: 100 μm.
2. Relative mRNA expression of *scgn* upon hypothalamus tissue in shControl and shScgn mice. N = 3 for each group. shControl: PVN Control-shRNA injection mouse. shScgn: PVN Scgn-shRNA injection mouse.
3. Plasma oxytocin concentrations of shScgn mice is decreased. N= 8 for each group. Data are presented as mean ± SD, P values were calculated using one-way ANOVA, Tukey's multiple comparisons test. **p < 0.01.
4. Schematic representation of sociability and recognition (three-chamber) test. Time spent interacting with a novel object or a novel mouse is shown for each genotype when injected with Control-shRNA EGFP and Scgn-shRNA EGFP AAV virus in PVN.
5. Representative heat maps showing the duration and location of shControl and shScgn mice during the 10- min social approach test. Warmer colors (red) indicate a greater amount of time spent exploring by the mice. “S1” and “O” represent the novel mouse and the novel object, respectively. shControl: PVN control-shRNA injection mouse.shScgn: PVN Scgn-shRNA injection mouse.
6. Bar chart showing chamber times spending interacted with a novel object or a novel mouse of shControl and shScgn mouse. S1: novel mouse. Center: center region in chamber. O: novel object. P values were calculated using one-way ANOVA, Tukey's multiple comparisons test. ***p < 0.001, *p < 0.05.
7. Bar chart showing sniffing times spending with an novel object or a novel mouse of shControl and shScgn mouse.P values were calculated using one-way ANOVA, Tukey's multiple comparisons test. ***p < 0.001.
8. Statistics of the social preference index (S1-O/total) from chamber time in (f). P values were calculated using T-test. Ns, no significant.
9. Statistics of the social preference index (S1-O/total) from sniffing time in (g). P values were calculated using T- test. Ns, no significant.

**Fig. S8.**


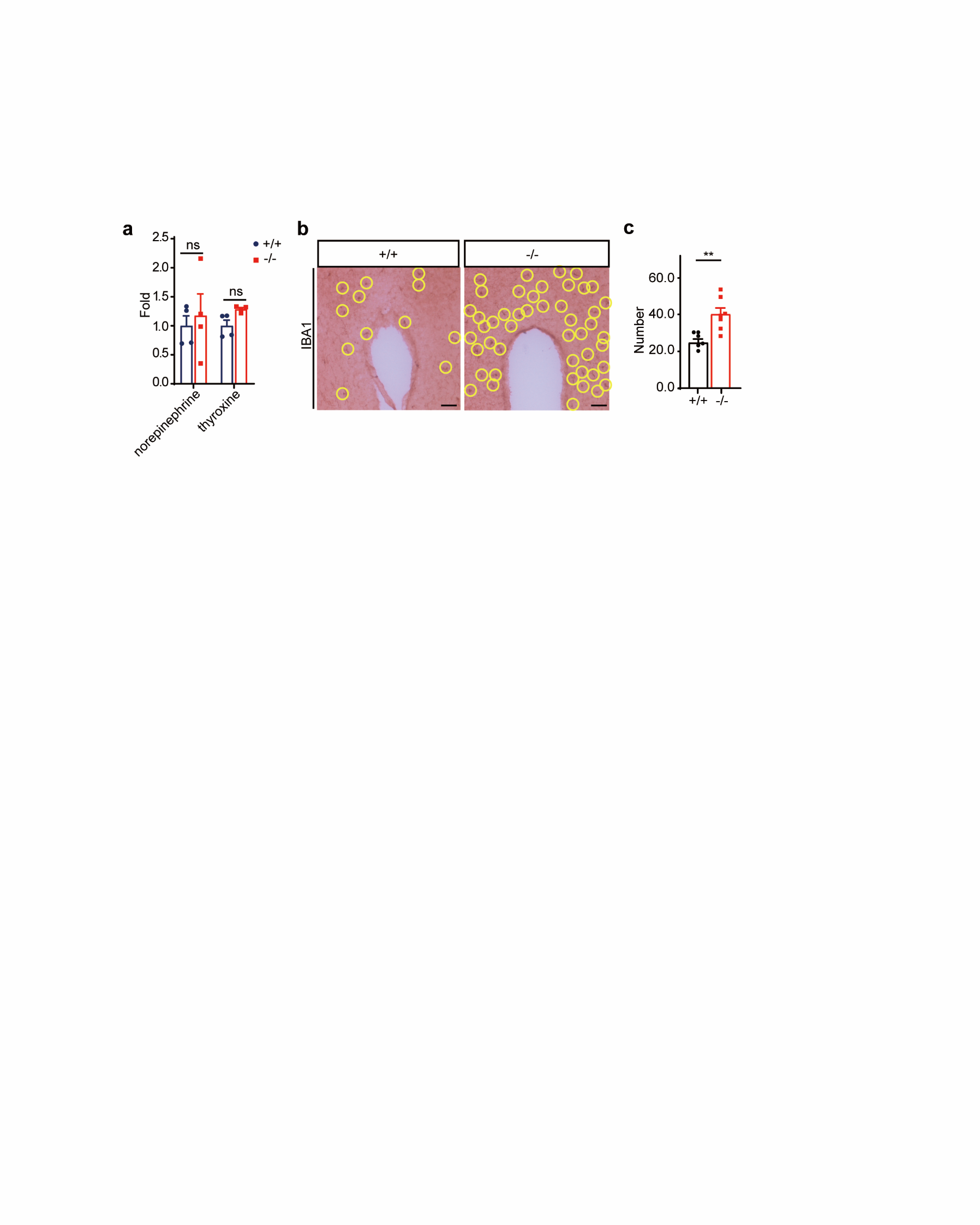


**Fig. S8 Depletion of SCGN in the PVN leads to the activation of microglia in mice.**

1. Depletion of SCGN did not impair the secretion of norepinephrine and thyroxine. N=4 for each group. Data are presented as mean ± SD, P values were calculated using T- test. ns, no significant.
2. Representative images of histochemical staining of IBA1^+^ microglia in the hypothalamus of *scgn*^+/+^ and *scgn*^-/-^ mice. *Scgn*^-/-^ mice showed a more dense distribution of IBA1^+^ microglia in *scgn*^-/-^ mice relative to that of the *scgn*^+/+^ mice. The circles show IBA1^+^ microglia. +/+: *scgn*^+/+^, −/−: *scgn*^-/-^. Scale bar: 50 μm.
3. Comparison of the total number of IBA1^+^ microglia in the hypothalamus of two groups of mice. N = 7 for each group. +/+: *scgn*^+/+^, −/−: *scgn*^-/-^. P values were calculated using T- test. **p < 0.01.

**Table. S1 Clinical diagnosis of people with *SCGN* mutation**

|  | | **ASD0010** | **ASD0014** | **NOTE** |
| --- | --- | --- | --- | --- |
| **Age** | | 3.2 years | 2 years |  |
| **Gesell development scale (Developmental Quotient score)** | DQ at motor domain | 58 | 58 |  |
|  | DQ at fine motor domain | 58 | 58 |  |
|  | DQ at adoptive behaviors domain | 58 | 58 |  |
|  | DQ at language development domain | 21 | 23 |  |
|  | DQ at personal social development domain | 39 | 39 |  |
|  | DQ total | 46 | 47 |  |
| **CARS** | | 43 | 41 | Severe（31-45） |
| **ABC** | | 68 | 67 | ASD≥53 |
| **CABS** | | 15 | 14 | ASD≥14 |

Note: CARS (Childhood Autism Rating Scale); ABC (Autism Behavior Checklist); CABS (Clancy Autism Behavior Scale)
